# Comparative attenuation of TiO₂ nanoparticle dose-dependent toxicities in lung, spleen, and blood by nanoencapsulated wheat germ oil in male rats

**DOI:** 10.1038/s41598-026-59659-5

**Published:** 2026-07-08

**Authors:** Nagat D. Kotb, Mai M. Ali, Marium M. Shamaa, Sabah G. El-Banna

**Affiliations:** 1 Division of Pharmaceutical Sciences College of Pharmacy, Arab Academy for Science, Technology and Maritime Transport, Alexandria, Egypt; 2 Department of Pharmaceutics, Division of Pharmaceutical Sciences, College of Pharmacy, Arab Academy for Science, Technology and Maritime Transport, Alexandria, Egypt; 3 Department of Biochemistry, Division of Clinical and Biological Sciences, College of Pharmacy, Arab Academy for Science, Technology and Maritime Transport, Alexandria, Egypt; 4 Biochemistry, Department of Environmental Studies, Institute of Graduate Studies and Research, Alexandria University, Alexandria, Egypt

**Keywords:** Titanium dioxide nanoparticles, Wheat germ oil-nanostructured lipid carrier, Oxidative stress, Lung-toxicity, Spleen-toxicity, Epidermal growth factor, Biochemistry, Biotechnology, Drug discovery, Nanoscience and technology

## Abstract

This study aimed to prepare and characterize nanoencapsulated wheat germ oil (WGO), to evaluate the dose-dependent toxicity of titanium dioxide nanoparticles (TiO_2_ NPs) in rat lungs, blood, and spleen, and to investigate the protective efficacy of both free and nanoencapsulated WGO, with particular emphasis on oxidative stress, immunotoxicity, and genotoxicity. WGO was nanoencapsulated and characterized for its colloidal properties, revealing desirable physicochemical attributes (spherical nanoparticles; size 173.9 ± 0.51 nm; zeta potential 14.4 ± 0.23 mV; PDI 0.39 ± 0.01) and strong in vitro antioxidant activity. To evaluate tissue injury, hematological parameters, oxidative stress biomarkers, and antioxidant enzyme activities were assessed. TiO_2_ NPs accumulated primarily in the lung and spleen, causing dose-dependent biochemical changes and immunomodulatory disruptions, such as increased TBARS and NO levels, DNA damage, and depletion of antioxidant enzymes. Histological and immunohistochemical analyses confirmed significant tissue damage. Co-administration of WGO mitigated these effects, with the nanoencapsulated form showing superior protection over the free form. Thus, nanoencapsulated WGO effectively counters TiO_2_ NP-induced pulmonary and splenic toxicity, likely via enhanced antioxidant and immunomodulatory actions, highlighting its promise as a therapeutic approach in nanotoxicology, occupational health, and food safety.

**Supplementary Information:**

The online version contains supplementary material available at 10.1038/s41598-026-59659-5.

## Introduction

Recently, nanotechnology has seen the introduction of engineered nanoparticles into many consumer and industrial products^1^, which has built significant commercial value but also raised public health concerns^2^. Among these, TiO_2_ NPs are the most produced; they are found in sunscreens, cosmetics^3^, and as a food additive (E171)^4^, leading to increased human exposure. The risk of chronic exposure may cause or worsen inflammatory and oxidative stress-related diseases, raising large-scale questions with major economic implications and adding a significant burden to global healthcare systems^5^**.** This public health issue is supported by growing toxicological evidence of the adverse biological effects of TiO_2_ NPs^6^**.** At the molecular level, TiO_2_ NPs have detrimental effects by creating a stressful environment rich in reactive oxygen and nitrogen species (ROS/RNS)^7^. Despite growing concerns regarding the safety of TiO_2_ NPs, regulatory agencies worldwide continue to hold conflicting positions regarding their use. While the International Agency for Research on Cancer (IARC) has classified TiO_2_ as a possible human carcinogen (Group 2B), and several authorities, including the European Union, have restricted or banned its use as a food additive, other countries, such as the United States, Canada, and Australia, continue to consider TiO_2_ safe under approved conditions. These discrepancies reflect the ongoing scientific uncertainty surrounding the potential health risks associated with TiO_2_ exposure. Moreover, the widespread use of TiO_2_ NPs in consumer products and pharmaceuticals increases the likelihood of human exposure, emphasizing the need for further studies to elucidate their toxicological effects and to identify effective strategies for mitigating TiO_2_ NP-induced tissue damage.

Historically assessed via inhalation^8,9^, the biokinetic trajectory of TiO_2_ NPs absorption presents a complex challenge to the splenic-pulmonary axis^10^. These NPs have particularly reactive surfaces, which lead to chemical reactions that break down phospholipids in cell membranes, impair mitochondrial function, trigger inflammation, damage DNA, and induce cell death^4^.. At the same time, the spleen, our body’s blood cell quality-control center, collects these particles in special compartments. This buildup can trigger a chain reaction, leading to damage in multiple organs and upsetting the immune system’s balance^11,12^. This damage doesn’t stop there; when red blood cells (RBCs) are exposed to this oxidative stress, they become compromised^13^ and start to release iron. This free iron can then be trapped in the spleen, leading to a shortage of usable iron in the body and, over time, can cause anemia^14^. Despite intraperitoneal (IP) administration circumventing direct pulmonary exposure, systemic translocation of TiO_2_ NPs can instigate secondary pulmonary toxicity. This route is also relevant to the growing biomedical applications of TiO_2_ NPs, including their investigation as drug delivery vehicles and sensitizers in cancer therapies^15,16^. Following IP injection, NPs access the systemic circulation, leading to their biodistribution and accumulation in distant organs, including the lungs and spleen. Within the pulmonary milieu, these translocated NPs can incite oxidative stress, inflammatory responses, and subsequent histopathological alterations. Consequently, alterations in epidermal growth factor (EGFR) expression serve as a molecular indicator of pulmonary tissue damage ensuing from systemic NP exposure^17,18^.

The expanding understanding of TiO_2_ NPs’ toxicity highlights a critical need for safe and effective mitigation. An optimal cytoprotective agent would be a natural, biocompatible compound exhibiting significant antioxidant and anti-inflammatory capacities, along with established human safety. Wheat germ oil is a good choice that stands out. Tocopherols (a form of vitamin E) and polyunsaturated fatty acids (PUFAs) are two of the many beneficial nutrients in WGO. These nutrients are known for their strong antioxidant properties^19^. They help neutralise harmful free radicals, which makes WGO a good choice for protecting the body from the stress that nanoparticles can cause. However, the therapeutic efficacy of lipophilic compounds like those in WGO is often limited by their poor aqueous solubility and low bioavailability^20^. Nanoencapsulation may significantly enhance its dispersibility, stability, cellular uptake, and facilitate more efficient delivery of the protective bioactive constituents to target intracellular sites^21^, potentially amplifying their ability to counteract the oxidative and inflammatory damage inflicted by TiO_2_ NPs^22,23^. Given the persistent concerns regarding TiO_2_ NP toxicity and the lack of effective protective interventions, further investigation of natural antioxidant-based approaches is warranted. In particular, limited information is available regarding the protective efficacy of nanoencapsulated wheat germ oil against TiO_2_ NP-induced splenic-pulmonary axis injury, highlighting the need for the present study. This study investigates the preventive efficacy of chitosan-coated nanostructured lipid carriers (NLCs) as a mucoadhesive, high-surface-area delivery platform. It demonstrates a robust approach to preserving the functional integrity of the splenic-pulmonary axis amid nanoparticle-induced systemic damage.

## Materials and methods

### Materials

Titanium dioxide NPs (50 ± 5 nm) were purchased from NanoTech Egypt Photo-Electronics Communication Center, El-Wahaat Road, City of 6 October, Al Giza, Egypt. WGO was purchased from Imtenan Company, Obour City, Egypt. Chemical reagents, including reduced glutathione (GSH), 1-chloro-2, 4-dinitrobenzene (CDNB), nicotinamide adenine dinucleotide phosphate (NADPH), Reduced Nicotinamide Adenine Dinucleotide (NADH), nitroblue tetrazolium (NBT), phenazine methosulfate (PMS), and thiobarbituric acid reactive substances (TBARS), were supplied by Sigma Chemical Company (Saint Louis, USA). Chitosan and cremophor RH-40 were purchased from Alpha Chemica Company (39 Melsa Bldgs., Ard El Golf, Heliopolis, Cairo, Egypt). Hemoglobin (Hb) assay kits (CAT. NO. MD-101106) were obtained from Mission Diagnostics (Inc., 333 Fiske St., Holliston, MA 01,746), nitric oxide (CAT NO. 2533), and catalase (CAT NO. CA 25 17) were obtained from Bio Diagnostic company (29 Tahreer St., Dokki, Giza, Egypt).

### Methods

#### Gas chromatography-mass spectroscopy of WGO

WGO was analyzed via GC–MS at the Institute of Public Health, Alexandria University, Egypt, using a Thermo Scientific Trace 1300 GC Ultra/ISQ QD Mass Spectrometer and Xcalibur 2.2 software. A 1 µL aliquot was injected, and chemical components were identified by comparing the GC eluent mass spectra with the MS database (test conditions are provided in the supplemental file).

##### Synthesis of the bioactive delivery platform

The fabrication of WGO-loaded NLCs was achieved through a melt-homogenization-sonication procedure^24^. A lipidic phase comprising solid and liquid bioactives was emulsified into an aqueous surfactant solution (Cremophor RH40) under high-shear conditions (Unidrive CAT1000 CAT Scientific) (probe sonicator; Vibra-Cell, USA). To ensure GIT absorption^25^, the resulting nanoparticles were surface-functionalized with a cationic chitosan coating^26^. The details of the formulations studied are listed in Table 1**.** Colloidal stability and physicochemical properties, including mean hydrodynamic diameter determined by DLS^27^, using Malvern Zeta Sizer Nano-ZS, UK, morphological uniformity assessed using TEM^28^**,** and Fourier-transform infrared spectroscopy (FTIR), were employed to investigate molecular compatibility among WGO and the excipients used in the optimized formulation using a Cary 630 FTIR spectrometer 4000–650 cm^−1^ (Agilent, USA). Finally, loading efficiency was evaluated via indirect ultrafiltration^29^**,** using the following equation:

$$EE\% = \frac{{{W_T} - {W_F}}}{{{W_T}}} \times 100$$

where W_T_ is the total amount of oil added to the NLCs, and W_F_ is the free unentrapped oil in the supernatant. In addition, stability of the nanoencapsulated WGO was assessed for particle size, PDI, and zeta potential following one month of storage at 4 °C in sealed amber vials^30^.

**Table 1:** Formulation and optimization of nanostructured lipid carrier loaded with WGO.

Formulation code	Homogenization (min)	Ultra sonication (min)	w/w % Oily phase : aqueous : SPAN			Water
			Cetyl alcohol: WGO (1:2)	Cremophor RH40	SPAN 80	
F1	10	-	3	6	1	To 10g
F2	10	-	3	6	2	
F3	10	-	3	3	2	
F4	10	-	3	3	1	
F5	10	2	3	3	1	
F6	10	5	3	3	1	
F7*	10	5	3	3	1	To 8g
F8* placebo	10	5	3	3	1	

* F7, F8 weight was completed to 10 g using chitosan solution.

#### In vitro antioxidant activity

The antioxidant potential of free wheat germ oil (WGO) and its nanoencapsulated form was evaluated using two complementary assays. Free radical scavenging activity was determined via the 1,1-diphenyl-2-picrylhydrazyl (DPPH) method^31^. Furthermore, the reducing capacity of the oil was measured using the ferric reducing antioxidant power (FRAP) assay^32^ (Details of each test condition are given in the supplemental file).

#### Animal study and dosing protocol

Sixty adult male rats (*Rattus norvegicus*), two months of age (146 ± 5 g), were obtained from the animal house, faculty of medicine, Alexandria University, Egypt, and acclimated for two weeks before the experiment. Then they were stratified into ten cohorts (n = 6) and housed in Universal galvanized wire cages at room temperature (22–25 °C) and in a photoperiod of 12 h/day. Animals were provided with a balanced commercial diet to evaluate the dose-dependent impact of intraperitoneally administered TiO2 NPs (5 and 25 mg/kg body weight)^33–35^**.** Briefly, TiO_2_ NPs were dispersed in normal saline to obtain the required concentration, then bath sonicated for 15 min (Branson_CPX2800). Prophylactic WGO (native or NLC-encapsulated) was administered via oral gavage at a dosage of 270 mg/kg body weight^36^**.** Following a subchronic exposure period (six-week duration, 3 days per week), anesthesia was induced with isoflurane inhalation, and biological samples were collected for comprehensive systemic analysis. The experimental protocol received ethical approval (ALEXU-IACUC; approval number AU14-230,614–2–4), and procedures were strictly adhered to international guidelines^37^. Details of the groups and treatments are presented in Table 2.

**Table 2:** Experimental animal design.

Group	Treatment	Dose- route
Group I	control	Saline- intraperitoneal
Group II	WGO	270 mg/kg body. Weight- oral
Group III	WGO-NLC	270 mg/kg body. Weight—oral
Group IV	Blank nanostructured lipid carriers	Oral
Group V	TiO_**2**_ NPs	5 mg/kg body. Weight Tid1- intraperitoneal
Group VI	Tid1 + WGO	Combination treatment
Group VII	Tid1 + WGO NLC	Combination treatment
Group VIII	TiO_2_NPs	25 mg/kg body. Weight Tid2- intraperitoneal
Group IX	Tid2 + WGO	Combination treatment
Group X	Tid2 + WGO NLC	Combination treatment

#### Organs pathophysiological assessment

Systemic integrity was evaluated through erythrocytic and leukocytic profiling of whole blood^38^. Blood was collected from the heart in heparinized tubes for the hematological study. Lung and spleen were isolated and washed with saline. Intact parts of the organs used for histological and immunohistological studies were kept in formalin (10%). Intact parts of the organs used for inductively coupled plasma were washed with saline and stored at −80 ºC till time of analysis. Tissue-specific peroxidative burden and nitro-oxidative stress were quantified in lung and spleen homogenates via nitric oxide (NO) and malondialdehyde (MDA) assays^39,40^, respectively. The functional status of the endogenous antioxidant network was mapped through the activities of glutathione reductase (GR), Glutathione S-Transferase (GST), superoxide dismutase (SOD), catalase (CAT), and glutathione peroxidase (GPx) activities^41^^42–45^, respectively, alongside reduced glutathione (GSH) levels^46^. Genomic stability was assessed using the DNA fragmentation assay for lung and spleen^47^ (details of each test are given in the supplemental file).

#### Titanium content in tissue using inductively coupled plasma

To quantify titanium levels, lung and spleen tissues were analyzed via ICP-ES Agilent^48^. Briefly, weighed samples underwent overnight pre-digestion (5 mL HNO_3_:1 mL H_2_O_2_​) followed by microwave-assisted digestion at 210 °C for 25 min. Once clear, the digests were filtered and adjusted to 10 mL using double-distilled water. The study utilized a quantification limit of 0.042 µg/g (details of the test are given in the supplemental file).

#### Histological and immunohistochemical studies

Parts of lung and spleen tissues were fixed in 10% formaldehyde solution, embedded in paraffin wax, and cut with a microtome for 5µ thick sections. The sections were stained with hematoxylin and eosin (H&E) stains and microscopically studied to investigate the histomorphological changes^49^**.** The immunohistochemical study to detect epidermal growth factor EGFR was performed according to^50^ (details of the test are given in the supplemental file).

#### Statistical analysis

To analyze the biological parameters, a one-way analysis of variance (ANOVA) was employed, followed by Tukey’s HSD multiple comparison test^51^**.**

## Results and discussion

### Gas chromatographic—mass spectroscopic identification of WGO

**Fig. 1:**
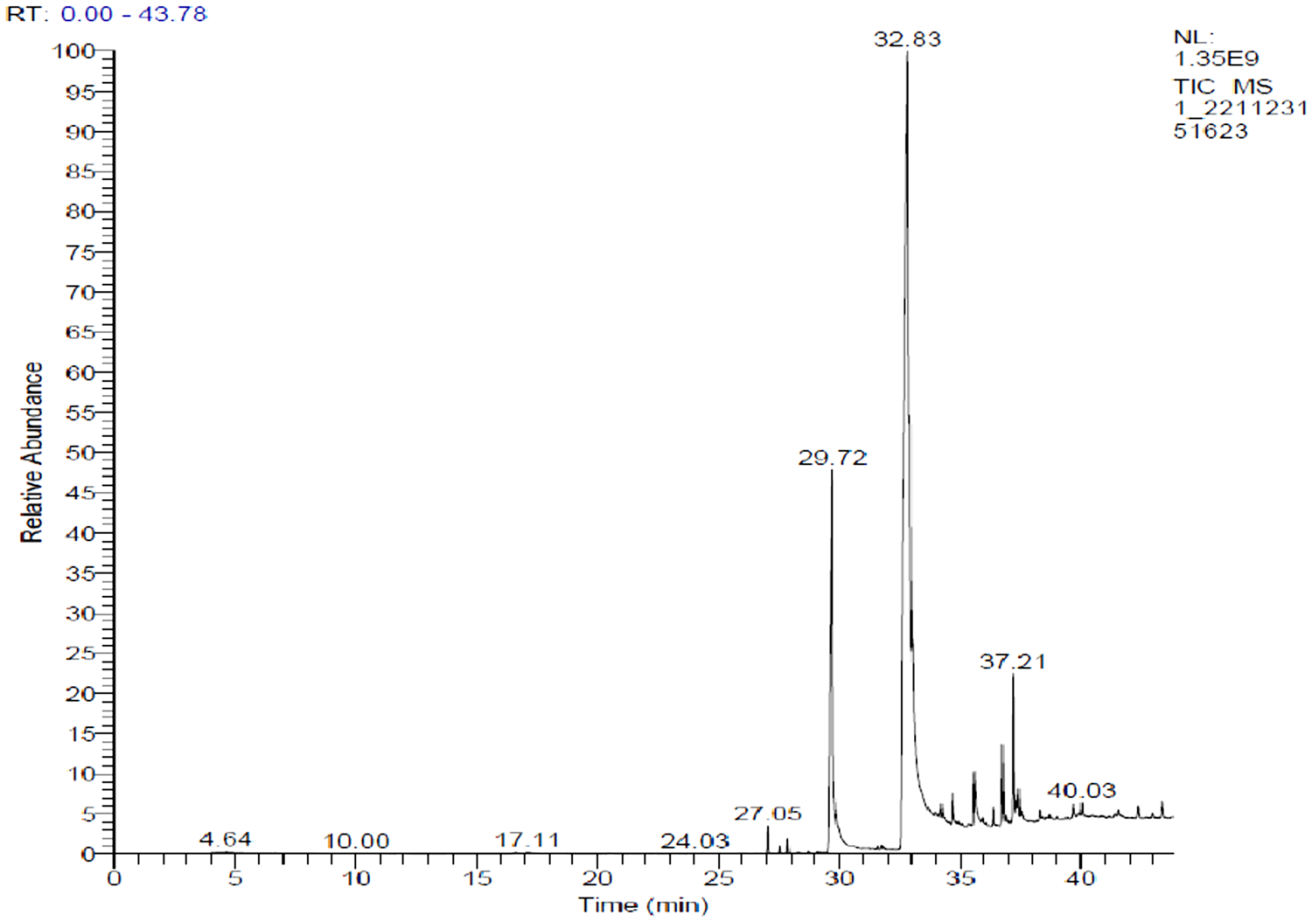
GC–MS analysis of the chemical composition of WGO.

The chemical composition of WGO employed in this investigation was evaluated using gas chromatography–mass spectrometry (GC–MS). The obtained results showed about 22 peaks in the GC–MS chromatogram Fig. 1, which indicates the presence of 22 peaks demonstrating the existence of 22 different compounds, by comparing the mass spectra of these molecules with the database of the mainlib, replib and NIST libraries. Table 3 shows the seven most prominent compounds that were identified in the analysis of WGO, with 9,12 Octadecadienoic acid represents (65.07%), n Hexadecanoic acid (16.45%), Oleic acid (5.73%) and 9,12 Octadecadienoyl Chloride (2.85%), and palmitoleic acid (2%) where the detected compounds accounted about 90% of the total amount. The obtained results indicate that WGO is characterized by the presence of a synergistic mixture of saturated and unsaturated fatty acids which makes it therapeutically effective, as reported in the literature^52–54^. The most prevalent compounds were.

**Table 3:** Gas chromatographic—mass spectroscopic analysis of wheat germ oil.

Compound Name	Molecular Formula	RT	Area %
9,12 Octadecadienoic acid (Z,Z) (Linoleic acid) (omega-6)	C_18_H_32_O_2_	32.83	65.07
n Hexadecanoic acid (Palmitic acid)	C_16_H_32_O_2_	29.72	16.45
Oleic acid (omega-9)	C_18_H_34_O_2_	33.02	5.73
9,12 Octadecadienoyl Chloride (Z,Z) (Linoleoyl chloride)	C_18_H_31_C_lO_	37.21	2.85
9Hexadecenoic acid (palmitoleic acid) (omega 7)	C_16_H_30_O_2_	35.60	2.00
9,12,15-octadecatrienoic acid (linolenic acid) (omega-3)	C_18_H_30_O_2_	37.35	0.48
3,7,11,15-tetramethylhexadec-2-en-1-ol (phytol, acetate)	C_22_ H_40_O_2_	27.05	0.42

Linoleic Acid (9,12 Octadecadienoic Acid) is a polyunsaturated omega-6 fatty acid (PUFAs), also known as omega-6, which offers excellent antioxidant activity, anti-mutagenic, and anti-tumor efficiency^55^**.** On the contrary, alpha-linolenic acid represent 0.48%, also known as omega-3, has a vital function in controlling lipid metabolism and protecting cardiovascular health^56^**.** Monounsaturated fatty acids, such as oleic acid, also known as omega-9, and palmitoleic acid, known as omega-7, are involved in maintaining homeostatic balance in the body by boosting immunity, increasing insulin sensitivity, and preserving vascular integrity^57,58^**.** Moreover, saturated fatty acid palmitic acid showed metabolic regulatory and anti-proliferative activities^59^**.** Besides fatty acid components, WGO also includes secondary metabolites such as phytol derivatives, like phytol acetate prevented oxidative stress and apoptosis, which also contribute to the total antioxidant activity of the oil^60,61^.

### Preparation and physicochemical characterization of WGO NLCs

#### Preparation of WGO NLCs

**Fig. 2:**
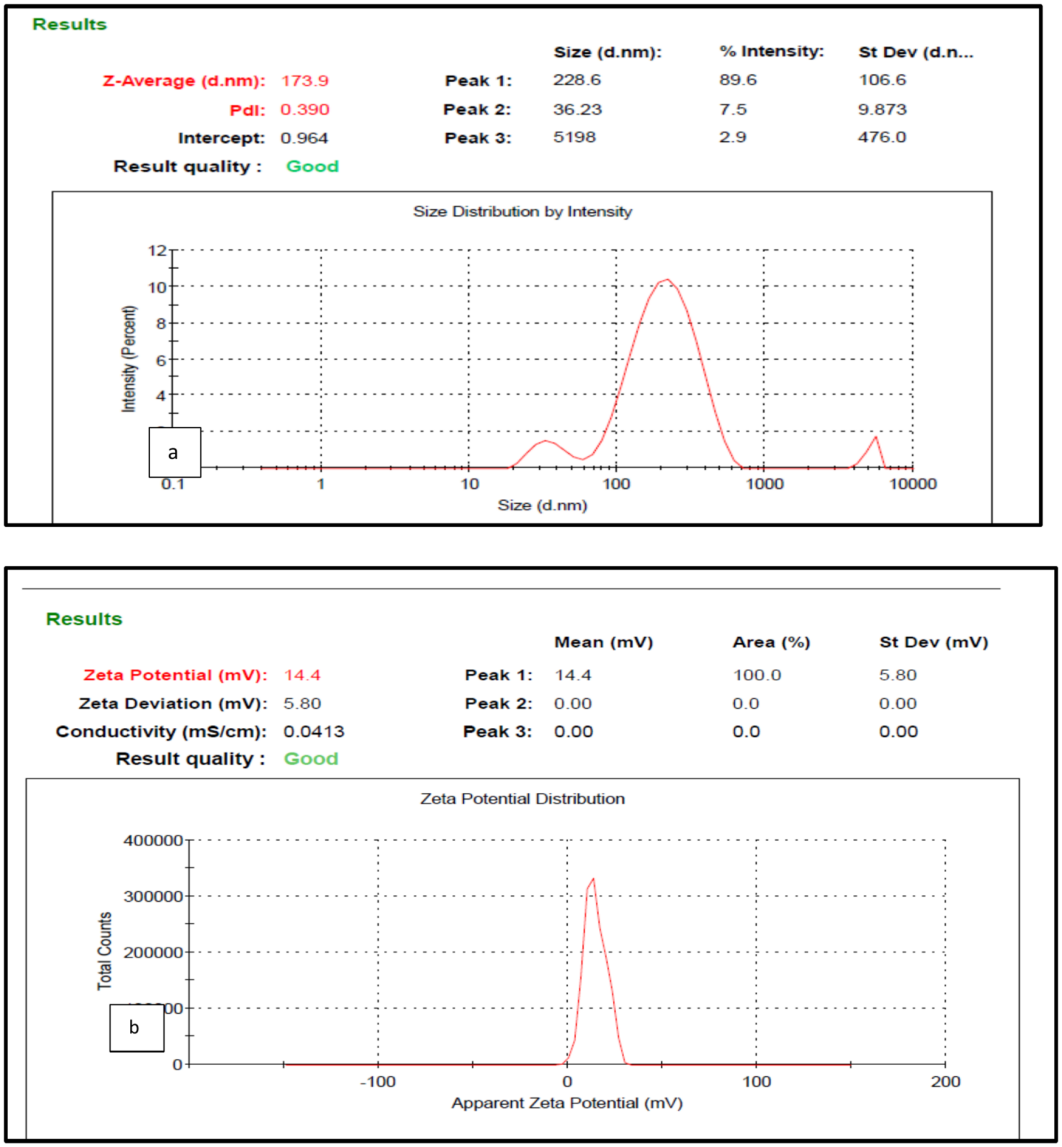

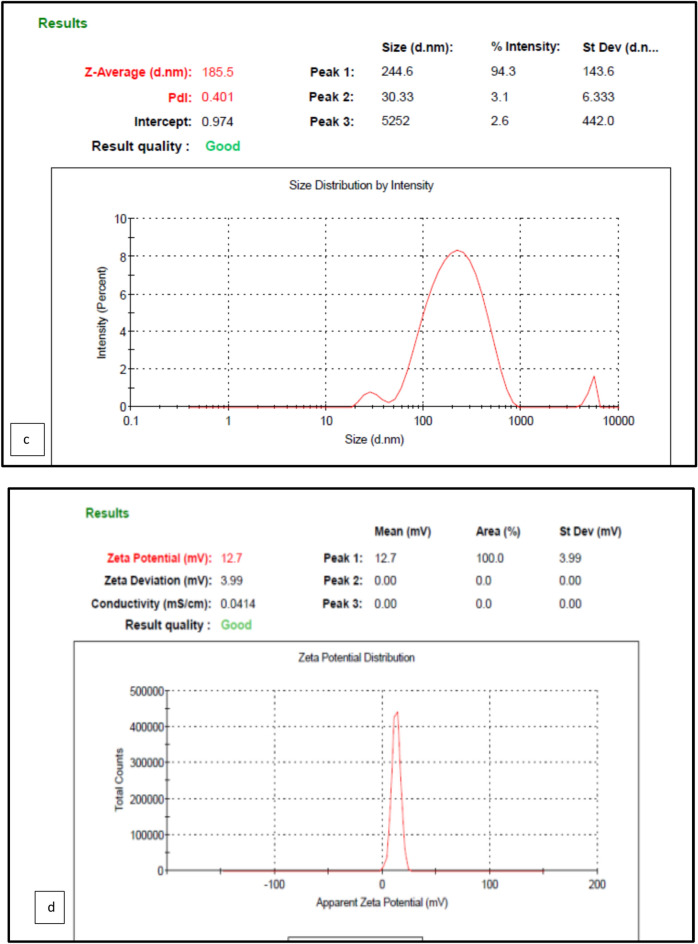
Physicochemical characterization of the optimized WGO-loaded NLC formulation: particle size distribution (**a**), zeta potential (**b**), particle size distribution after one month of storage at 4 °C (**c**), and zeta potential after one month of storage (**d**) at 4 °C.

Results of NLC formulations prepared are shown in Table 4 and Fig. 2a, b. In addition, the result of the stability study indicates that the nanoencapsulation is physically stable under refrigeration at 4 °C up to one month with slight increases in particle size (185.5 ± 0.03) and zeta potential (+ 12.7 ± 0.021). Overall, the formulation shows acceptable stability for storage and potential use Fig. 2c,d. Wheat germ oil was nanoencapsulated using a nano-delivery system, which was chosen because it was reported to have better delivery and bioavailability than free oil^20,23^. Nanostructured lipid carrier (NLC) was chosen instead of nano-emulsion because it is physically more stable, which can better preserve the valuable polyunsaturated constituents of WGO^62^. Cetyl alcohol was used as the solid lipid to avoid prolonged exposure to heat during preparation, which can be detrimental to the constituents of the oil^63,64^.

**Table 4:** Physicochemical properties of WGO NLCs.

Code	Oily phase: aqueous phase: SPAN % w/w	Condition (min)		Colloidal properties		
		Homogenization	Ultra sonication	Size	PDI	Zeta potential (Mv)
F1	3:6:1	10	-	182.8 ± 1.50	0.667 ± 0.01	−20.2 ± 0.17
F2	3:6:2	10	-	220.7 ± 0.69	0.595 ± 0.0	−10.6 ± 0.17
F3	3:3:2	10	-	203.6 ± 0.58	0.478 ± 0.02	−17.2 ± 0.17
F4	3:3:1	10	-	192.0 ± 0.23	0.453 ± 0.0	−23.5 ± 0.29
F5	3:3:1	10	2	198.5 ± 0.64	0.437 ± 0.02	−40.3 ± 0.12
F6	3:3:1	10	5	189.0 ± 0.29	0.334 ± 0.01	−55.0 ± 0.29
F7*	3:3:1	10	5	173.9 ± 0.51	0.39 ± 0.01	14.4 ± 0.23
F8* placebo	3:3:1	10	5	185.9 ± 0.40	0.395 ± 0.0	10.8 ± 0.35

The results are expressed as (Mean ± SE,n = 3).

##### Effect of span

Results presented in Table 4 show that increasing Span 80 was accompanied by an increase in the NLC particle size and a decrease in homogeneity, reflected in the high PDI. This may be attributed to the lipophilic nature of Span 80, which increases the viscosity of the lipid droplets during preparation. In addition, increasing Span 80 leads to a decrease in zeta potential, indicating lower colloidal stability. This may be explained by the structure of Span 80, which may mask the charge present on the droplet surface^65^. Increasing Span 80 content leads to larger niosomes, affected stability.

##### Effect of cremophor RH40

The formulations with 3% (w/w) Cremophor had smaller mean particle sizes and lower PDI values when compared with those formulations containing 6% (w/w) (F2/F3 vs. F1/F4). This may be related to the optimal surfactant-to-oil ratio, which at 3% (w/w) Cremophor is sufficient to reduce interfacial tension efficiently and stabilize oil droplets. Conversely, larger particle sizes were observed for formulations containing 6% (w/w) Cremophor, and this may be related to surfactant concentrations that are above a certain critical level. The presence of highly viscous liquid crystalline phases at these concentrations may increase internal viscosity, hindering efficient oil droplet disruption. Apart from particle size, another important parameter that influences the stability and interactions between colloids and cells is zeta potential, a parameter that has significant implications for cellular interactions^66^**.** The results suggest that zeta potential increases with decreasing concentrations of Cremophor, potentially related to a decrease in the screening effect that is characteristic of nonionic surfactants^67^**.** Despite the slight increase in particle size, formulation F4 was selected for subsequent studies due to its higher homogeneity and stability.

##### Effect of sonication time

The effect of ultrasonication time was studied to improve the colloidal characteristics of formulation F4. The increase in ultrasonication time from zero to five minutes did not significantly affect particle size but resulted in an improvement in homogeneity and colloidal stability of the prepared formulations (F5 and F6) in contrast to homogenization time alone (F4). The addition of sonication energy to the NLC dispersions caused the breakup of coarse emulsion droplets into nanoemulsion droplets, resulting in a decrease in particle size distribution and homogeneity as seen from PDI results. This result is consistent with previously reported data^68^. Accordingly, formulation (F6) was selected for further study.

##### Effect of chitosan coating

As NLCs loaded WGO was to be given through the oral route, surface coating with a mucoadhesive polymer like chitosan was selected to coat the selected formulation F6^69^. reported that chitosan improves oral absorption, bioavailability of drugs, and nanoformulations. The coated formulation F7 showed a lower particle size and homogeneity than the uncoated formulation. In addition, the zeta potential changed from (−55 to + 12.7 mV), indicating successful coating of the NLC with the cationic polymer chitosan. This result agrees with^70^**.**Additionally^66^, demonstrated that a positive zeta potential on the nanoparticle surface promotes their cellular internalization and accumulation. The high absolute zeta potential values observed in the present study indicate good colloidal stability, as electrostatic repulsion between particles reduces aggregation during storage and administration. Following chitosan coating, the conversion of the zeta potential from − 55 mV to + 12.7 mV not only confirmed successful surface modification but may also enhance mucoadhesion and interaction with negatively charged epithelial cell membranes. Such interactions can facilitate nanoparticle uptake and improve the oral bioavailability of the encapsulated oil, making the formulation more suitable for drug delivery applications.

#### Entrapment efficiency (EE %) of NLC loaded with wheat germ oil

The selected coated NLC loaded with WGO (F7) showed an average encapsulation efficiency of 99.65% ± 0.05, which may be explained by the WGO’s sufficient solubility in the lipid matrix as well as its hydrophobic character, which results in less WGO migrating into the aqueous phase. This result agrees with previous studies^71^.

#### Morphological characterization via transmission electron microscopy (TEM)

**Fig. 3:**
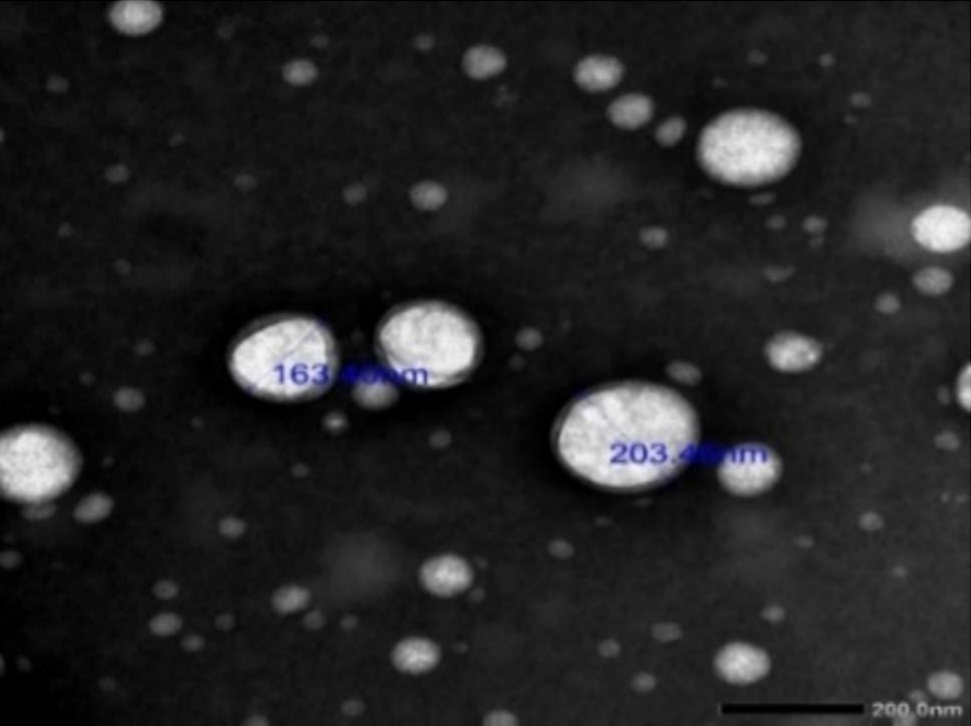
Transmission electron microscopy (TEM) analysis of WGO-loaded nanostructured lipid carriers (NLCs) of optimum formulation F7 (JEOL Ltd., USA). Images were acquired at 40,000 × magnification with an accelerating voltage of 120 kV.

The TEM results for the optimum formulation F7, shown in Fig. 3, indicated that the WGO-loaded nanoparticles were homogeneous, spherical, and well-defined due to the coating layer, with an average size comparable to that obtained in the dynamic light scattering (DLS) study. The observed difference between TEM-derived particle size and DLS hydrodynamic diameter is expected due to the fundamental differences in measurement principles. TEM analysis provides the actual core size of the nanoparticles in a dehydrated state under vacuum conditions, whereas DLS measures the hydrodynamic diameter of particles dispersed in solution, which includes the solvation layer, possible surface coating effects, and slight aggregation in the colloidal state^72^.

#### FTIR

**Fig. 4:**
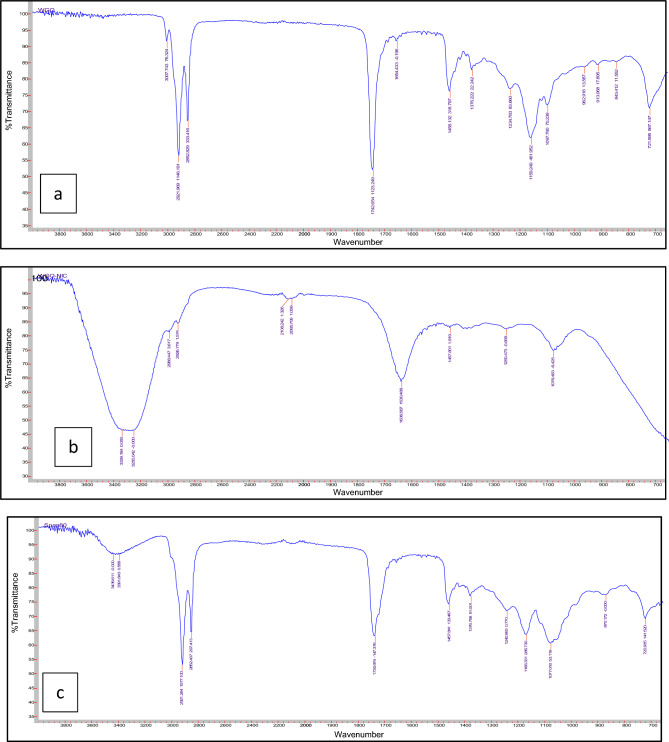

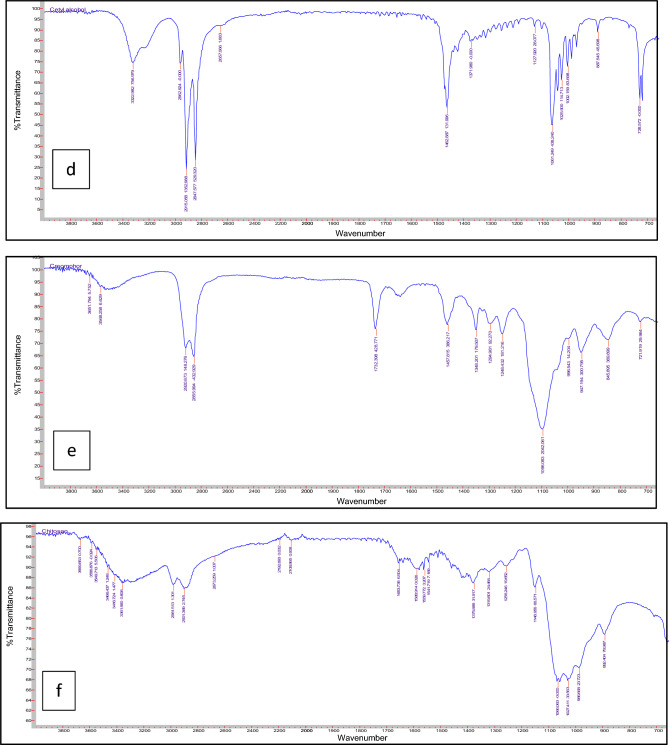
Fourier-transform infrared (FTIR) spectra of wheat germ oil (WGO), nanoencapsulated wheat germ oil (WGO-NIC), and formulation components. (**a**) WGO, (**b**) WGO-NIC, (**c**) Span 80, (**d**) cetyl alcohol, (**e**) Cremophor, and (**f**) chitosan.

FTIR measurements provide information on the potential functional groups of the biomolecules present in WGO and its nanoformulation^73^. As shown in Fig. 4**,** pure WGO exhibited characteristic absorption bands at 3007 cm⁻^1^ (═C–H stretching) reflecting the presence of unsaturated fatty acids, 2922 and 2853 cm⁻^1^ (aliphatic C–H stretching), and the strong absorption at 1743 cm⁻^1^ corresponding to the ester carbonyl group of triglycerides^74,75^. The obtained results of FTIR analysis of WGO are consistent with a previous study^76^. Chitosan displayed a broad O–H/N–H stretching band around 3362 cm⁻^1^ and characteristic amide bands at 1654 and 1589 cm⁻^1^^77,78^. In the optimized WGO-NLC formulation, broadening of the O–H/N–H region (3339 cm⁻^1^) together with attenuation and overlap of the characteristic WGO carbonyl band and the appearance of a dominant band at 1637 cm⁻^1^ suggest intermolecular interactions between WGO, the lipid matrix, and the chitosan coating. These changes are consistent with hydrogen-bond formation and electrostatic interactions within the nanostructured carrier system. Furthermore, the absence of additional bands indicates that no chemical degradation or covalent modification of WGO occurred during formulation, supporting preservation of its chemical integrity. The FTIR findings, together with the high encapsulation efficiency, positive zeta potential, and TEM observations, provide evidence for the successful formation and stabilization of the chitosan-coated WGO-NLC system.

#### Antioxidant activity DPPH and FRAP

Persistent generation of high-level reactive oxygen species (ROS) is a significant cause of chronic inflammation in biological systems. Phytoconstituents like flavonoids, fatty acids, and polyphenols have antioxidant properties that can counteract the ROS effects^79^.

##### Diphenyl-2-picrylhydrazyl (DPPH)

**Fig. 5:**
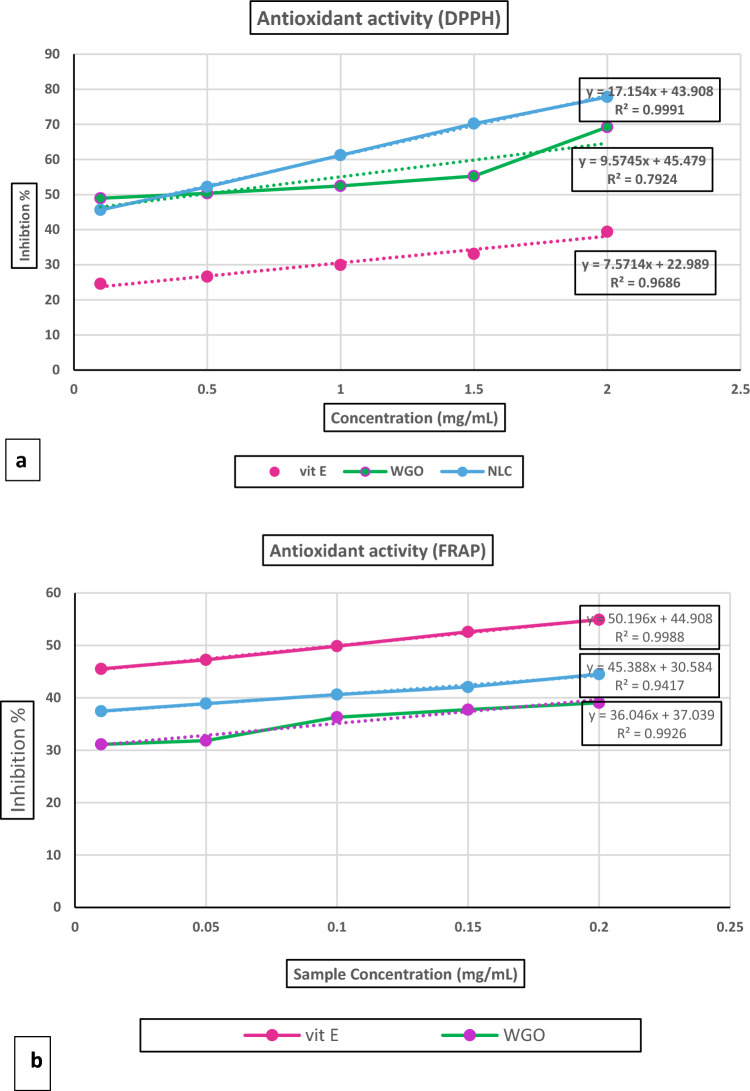
Antioxidant activity DPPH IC50 (**a**), FRAP EC50 (**b**) of WGO and WGO NLCs as compared with vitamin E.

The antioxidant capacity of WGO was characterized by a maximal DPPH inhibition of 66.36%, consistent with the ranges reported by^80,81^**.** While the Vitamin E standard achieved a higher total inhibition (95.69%) at peak concentrations, the WGO formulations demonstrated significantly higher efficiency at lower doses. This is evidenced by the IC_50_ values as shown in Fig. 5a**,** where WGO-NLC (0.355 mg/mL) and free WGO (0.47 mg/mL) outperformed Vitamin E (3.56 mg/mL). According to the classification by^82^, all samples reside in the ‘extremely strong’ antioxidant category. In addition, the superior efficiency of the WGO-NLC formulation suggests that nanostructured lipid encapsulation optimizes the delivery and stability of bioactive lipids. Owing to the higher available surface area per unit volume, the NLC system facilitates more effective radical scavenging at lower concentrations compared to both the free oil and the conventional Vitamin E standard.

##### Ferric reducing antioxidant power capacity (FRAP)

One of the most useful techniques that is used for assessing an antioxidant’s potency is FRAP, which describes the ability of a sample to donate an electron or hydrogen ion to convert ferric iron (III) to ferrous iron (II). Elevated ferric reducing antioxidant power signifies a more potent antioxidant activity and a greater ability of the sample to donate electrons or hydrogen ions. Consequently, corresponds to greater antioxidant activity in the sample^83^**. **Figure 5b represents the FRAP EC50 of WGO, WGO NLC, and vitamin E (35.95, 42.79 and 10.15) %, respectively. The obtained results showed that the EC50 of WGO loaded into NLCs was significantly higher than that of WGO in its free form and vitamin E. The same results were obtained by the study of^84^, which revealed that defatted wheat germ demonstrated antioxidant properties in various assays, including DPPH, ABTS, and FRAP. Similarly^85^, demonstrated that extrusion of pure wheat bran and heat treatment of wheat germ significantly enhanced antioxidant capacity (DPPH and FRAP) and increased phenolic acid and flavonoid content, facilitating their use in functional foods. These findings support our results, which showed that NLCs loaded with WGO exhibited superior antioxidant activity compared to the free oil.

### Animal study

#### Anthropometric measurements

**Fig. 6:**
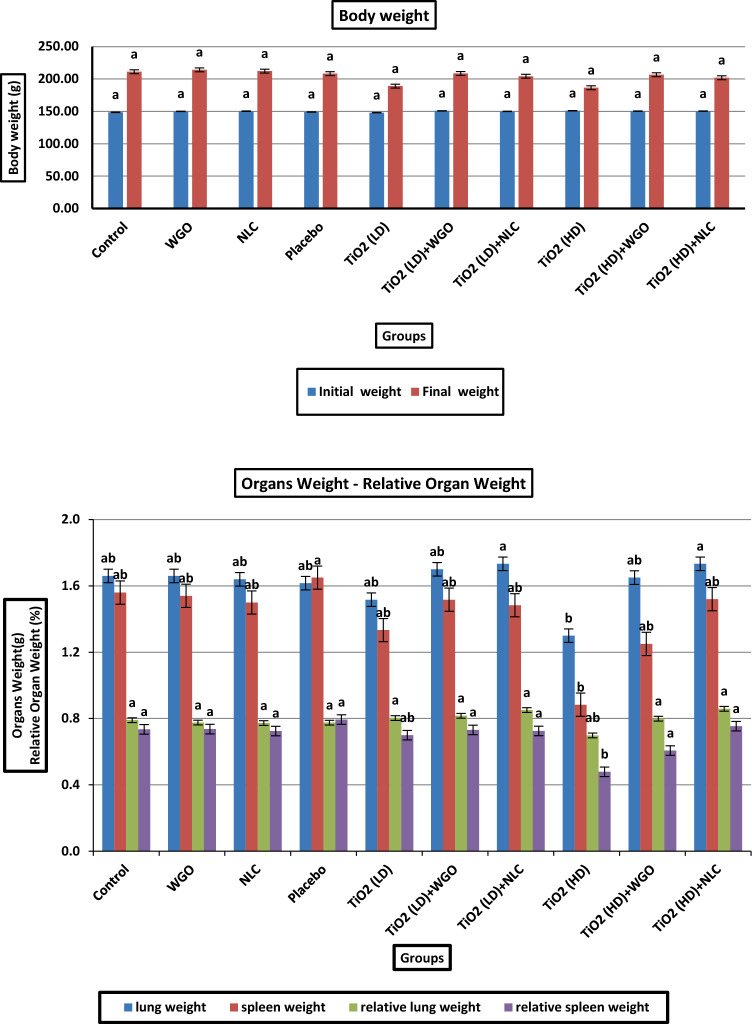
Effects of wheat germ oil and its nanoformulation on the titanium dioxide nanoparticles-induced toxicity on the body weight of male rats. The results are expressed as (Mean ± SE,n = 6). ^abcd^ Different superscript letters indicate statistically significant differences between groups (*p* < 0.05). TiO_2_ (LD); low dose of titanium dioxide nanoparticles (5 mg/Kg body weight), TiO_2_ (HD); high dose of titanium dioxide nanoparticles (25 mg/Kg body weight), WGO; dose of wheat germ oil (270 mg/Kg body weight), NLC; dose of wheat germ oil loaded nanostructured lipid carrier (270 mg/Kg body weight), Placebo; blank of NLC.

Investigating baseline physiological parameters like body weight, organ weight, and relative organ weight revealed that oral administration of placebo and free or nanoencapsulated WGO forms to healthy male Wistar rats had no significant impact, as shown in Fig. 6**,** aligning with previous studies^86^. This result further confirms that the WGO formulations are inert. Subsequent exposure to both levels of TiO_2_ NPs in a similar fashion did not reveal any notable differences in the terminal body weights of male rats, which is consistent with previous studies indicating that body weight effects are only expected at very high levels of TiO_2_ NPs^87,88^. However, absolute spleen weight showed greater sensitivity as an endpoint, where high-dose TiO_2_ nanoparticle exposure resulted in a statistically significant decrease or alteration in spleen weight, despite no such effects being observed for low-dose exposure. Such an outcome suggests possible hematological toxicity, as also concluded by^89^. In addition, relative organ weights were unaffected in all the TiO_2_ NPs-exposed groups.

Lastly, the simultaneous administration of both free and nanoencapsulated WGO formulations significantly reduced the adverse effects on absolute organ weights, indicating a protective effect against organ toxicity caused by TiO_2_ NPs. Although the intervention was effective, a residual effect was still apparent at the high-dose TiO_2_ (HD) relative to the control. Restoring organ weight is difficult even at high doses of a toxic substance. In conclusion, these findings suggest that while body weight is a less sensitive parameter, absolute organ weights, particularly the spleen, are susceptible to the toxic effects of TiO_2_ NPs, while both free and nanoencapsulated WGO formulations are a promising intervention strategy.

#### Titanium accumulation content in tissue homogenates of male rats

ICP-MS results confirmed significant tissue accumulation of Titanium (Ti) in male Wistar rats following TiO_2_ NPs exposure (10.96 µg/g in lung and 12.16 µg/g in spleen tissues in the high TiO_2_ NPs group). Detection of Titanium in the control spleen (3.26 µg/g) samples suggests potential environmental exposure, which is possible given the widespread industrial use of TiO_2_ NPs^90,91^. Co-administration of free and nano-encapsulated WGO significantly lowered tissue titanium levels. These findings confirm that TiO_2_ NPs can circulate systemically and accumulate within vital organs^92^ like the lung and spleen, consistent with previous reports^88,93^. While free WGO eliminated titanium from lung tissue and drastically reduced it in the spleen to (0.27 µg/g tissue), nanoencapsulated WGO completely prevented detectable titanium accumulation in both lung and spleen tissues. These findings collectively demonstrate that both free and, particularly, nanoencapsulated WGO can offer a potent strategy to mitigate titanium tissue accumulation and associated TiO_2_ NPs induced organ toxicity.

#### Hematological study

**Fig. 7:**
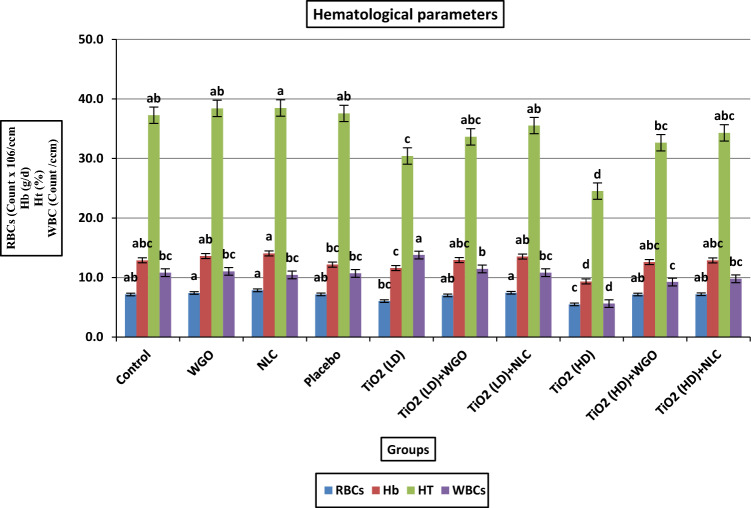
Effects of wheat germ oil and its nano-formulation on the titanium dioxide nanoparticles-induced toxicity on RBCs, Hb, Ht and WBCs in blood of male rats. The results are expressed as (Mean ± SE,n = 6). ^abcd^ Different superscript letters indicate statistically significant differences between groups (p < 0.05). TiO_2_ (LD); low dose of titanium dioxide nanoparticles (5 mg/Kg body weight), TiO_2_ (HD); high dose of titanium dioxide nanoparticles (25 mg/Kg body weight), WGO; dose of wheat germ oil (270 mg/Kg body weight), NLC; dose of wheat germ oil loaded nanostructured lipid carrier (270 mg/Kg body weight), Placebo; blank of NLC.

Hematological profiling serves as a sensitive indicator of the systemic internal environment, reflecting the functional integrity of the splenic-pulmonary axis under toxicological insult^94,95^. As illustrated in Fig. 7**,** the free or nanoencapsulated WGO and placebo groups (given nanoencapsulated formulation without WGO) showed parity to the control group, indicating both safety and biocompatibility that the oil and the nanostructured lipid carrier system.

Exposure to TiO_2_ NPs resulted in a dose-responsive effect on erythrocytes, as indicated by a significant reduction in RBC count, hemoglobin concentration, and hematocrit values. The rationale for the alterations in blood parameters following exposure to TiO_2_ NPs is most likely due to the systemic distribution of NPs in the body. The aggregation of TiO_2_ NPs in the extensive pulmonary capillary beds most likely induces a burst reaction, which in turn increases lipid peroxidation of erythrocyte membranes. The compromised erythrocytes are then removed from circulation by the mononuclear phagocyte system in the spleen, resulting in a dose-responsive effect in RBC count, hemoglobin, and hematocrit values. The reduction in erythrocyte parameters, therefore, reflects a systemic effect of TiO_2_ NPs exposure. The findings of this study are in agreement with previous reports^96,97^. Meanwhile, a dose-dependent divergent response was noted in the leukocytic population, which points to the presence of a systemic strain condition. Leukocytosis in the lower dose of TiO_2_ NPs was attributed to the physiological response to nanoparticle-induced acute inflammation. However, the onset of leukopenia in the higher dose of TiO_2_ NPs points to severe systemic exhaustion, where oxidative insult results in depletion of the circulating immune cells. Changes in leukocyte counts may reflect dose-dependent immune modulation in response to TiO_2_ exposure.

The administration of free WGO was noted to have significant protective effects on TiO_2_ NP-induced hematotoxicity, as manifested by the normalization of erythroid and leukocyte parameters in both doses. Notably, the nano-encapsulated WGO was found to be more effective, normalizing all parameters to levels statistically equivalent to those of the healthy controls. This protective effect can be attributed to the bioactive ingredients of WGO, which are known to have antioxidant and anti-inflammatory properties, which may also stabilize erythrocyte membranes and erythropoiesis^98–100^. The enhanced therapeutic effect of the nanostructured lipid carrier (NLC) was attributed to the optimized design, which was also discussed above^101,102^. These findings underline the potential of nanoencapsulation to increase the bioavailability and therapeutic efficiency of bioactive oils, providing a robust systemic shield against nanoparticle-induced oxidative damage.

#### Effects on oxidative stress and genotoxicity

**Fig. 8:**
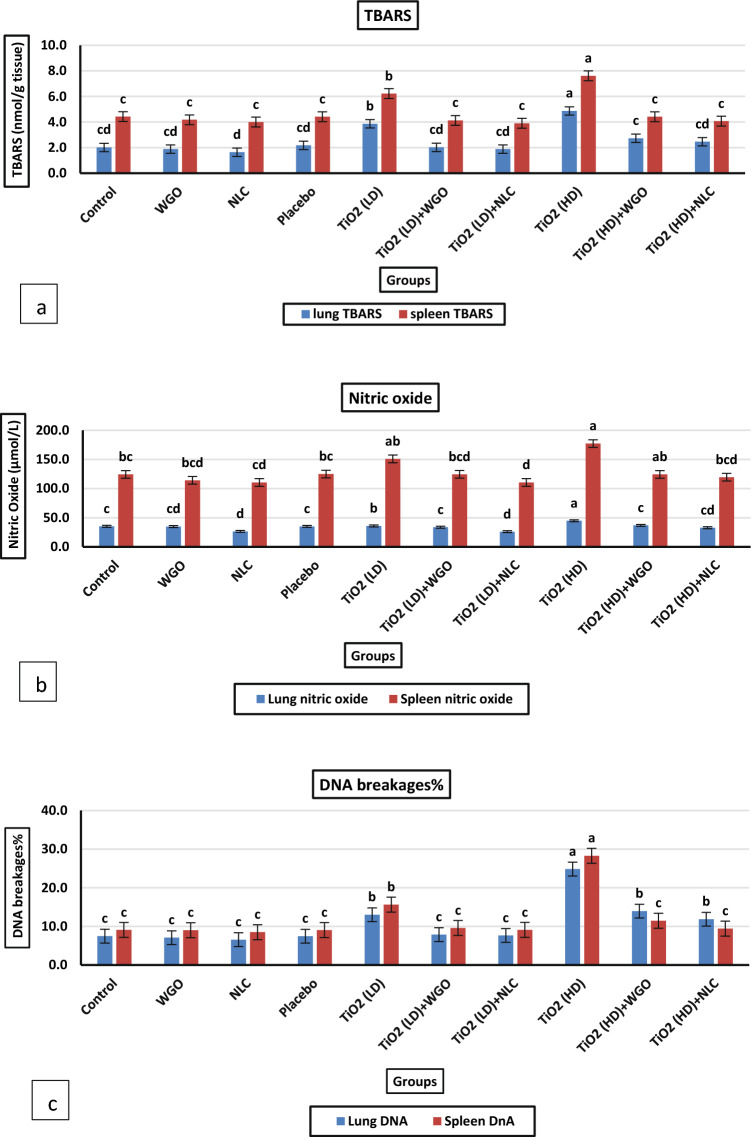
Effects of wheat germ oil and its nano-formulation on the titanium dioxide nanoparticles induced toxicity on TBARS (**a**), nitric oxide level (**b**) and DNA breakages (**c**) in lung and spleen homogenates of male rats. The results expressed as (Mean ± SE,n = 6). ^abcd^ Different superscript letters indicate statistically significant differences between groups (*p* < 0.05). TiO_2_(LD); low dose of titanium dioxide nanoparticles (5 mg/Kg body weight), TiO_2_(HD); high dose of titanium dioxide nanoparticles (25 mg/Kg body weight), WGO; dose of wheat germ oil (270 mg/Kg body weight), NLC; dose of wheat germ oil loaded nanostructured lipid carrier (270 mg/Kg body weight), Placebo; blank of NLC.

In a state of oxidative stress, a physiological imbalance occurs through the accumulation of ROS that exceeds the capability of the antioxidant defense mechanisms, culminating in lipid peroxidation (LPO)^103^. Malondialdehyde (MDA), measured via the thiobarbituric acid reactive substances (TBARS) assay, is a definitive biomarker of LPO, which is a result of the oxidative degradation of polyunsaturated fatty acids, which are vital for maintaining cellular membrane fluidity^104,105^. Exposure to TiO_2_ NPs induced dose-dependent oxidative and nitro-oxidative stress in both lung and spleen tissues, as evidenced by significant increases in TBARS and nitric oxide (NO) levels, along with elevated DNA strand breaks. The lung showed higher TBARS and NO levels, whereas DNA damage was slightly more pronounced in the spleen. These biochemical alterations were accompanied by systemic changes consistent with oxidative injury, as shown in Fig. 8a, b, c.

The observed increases in LPO and NO suggest enhanced reactive oxygen and nitrogen species generation following TiO_2_ exposure^34,106^. This pro-oxidant environment is likely involved in the observed genomic instability, as reflected by increased DNA fragmentation in both organs, consistent with oxidative stress–mediated cellular injury. These results are consistent with a large body of evidence^107–109^. Nanoparticle exposure was associated with hematological alterations suggestive of erythrocyte damage and subsequent disturbances in iron homeostasis. Oxidative stress generated by TiO_2_ nanoparticles may compromise erythrocyte membrane integrity, leading to hemoglobin release and alterations in hematological indices. The released iron can subsequently be sequestered by macrophages as a protective mechanism to limit iron-catalyzed oxidative reactions. This response is characteristic of functional iron deficiency or anemia of inflammation, in which iron remains stored within reticuloendothelial cells but becomes less available for erythropoiesis^110,111^. Such mechanisms may contribute to the reductions in hemoglobin concentration, erythrocyte count, and hematocrit observed in the TiO_2_ NP-exposed groups; however, the underlying mechanisms require further investigation.

Additionally, TiO_2_ NPs trigger a ‘nitro-oxidative’ burst, creating highly reactive molecules capable of LPO of the nuclear membrane, penetrating the nuclear envelope and attacking the deoxyribose backbone, leading to the cleavage of phosphodiester bonds and the formation of single- and double-strand breaks^112^. These observations align with a growing body of evidence of the ability of TiO_2_ NPs to compromise genomic stability^113–115^ and are supported by epidemiological data^116^ identifying elevated oxidative DNA damage in workers occupationally exposed to TiO_2_ NPs.

Co-administration of WGO attenuated these alterations in a dose-dependent manner. Free WGO partially reduced TBARS, NO levels, and DNA damage, while nano-encapsulated WGO produced a more pronounced effect, restoring these parameters close to control levels in both lung and spleen tissues. In addition, WGO-NLC-treated groups showed values that were not significantly different from controls in several endpoints, indicating improved efficacy of the nano-formulation. The enhanced performance of the nano-encapsulated formulation may be related to improved delivery and bioavailability compared with free oil, resulting in more effective mitigation of oxidative and genotoxic stress^101,102^. These observations are consistent with previous studies that have shown that nano-emulsified WGO is nephroprotective, reducing malondialdehyde levels^20^. The protective effect is attributed to its antioxidant activity, which reduces nitro-oxidative stress and preserves cellular integrity, thereby limiting DNA fragmentation. Moreover, the integration of WGO constituents with the phospholipid double layers of cellular and erythrocyte membranes creates a structural antioxidant layer, which maintains membrane integrity in the face of oxidative chain reactions consistent with previous studies^117^^118,119^^[,120^.

#### Effects on antioxidant enzymes

**Fig. 9:**
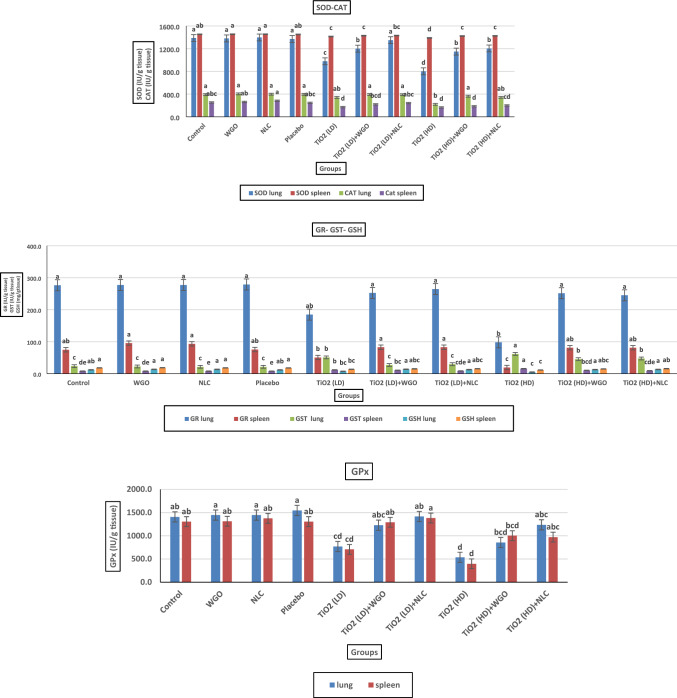
Effects of wheat germ oil and its nano-formulation on the titanium dioxide nanoparticles-induced toxicity on antioxidant enzymes of lung and spleen homogenates of male rats. The results are expressed as (Mean ± SE,n = 6). abcd Different superscript letters indicate statistically significant differences between groups (p < 0.05). TiO2(LD); low dose of titanium dioxide nanoparticles (5mg/Kg body weight), TiO2(HD); high dose of titanium dioxide nanoparticles (25mg/Kg body weight), WGO; dose of wheat germ oil (270mg/Kg body weight), NLC; dose of wheat germ oil loaded nanostructured lipid carrier (270mg/Kg body weight), Placebo; blank of NLC.

The state of endogenous antioxidant systems, as shown in Fig. 9**,** is considered a definitive marker of systemic redox imbalance caused by TiO_2_ nanoparticles. The results show that there is a significant, dose-related depletion of the main enzymatic defense pool, as reflected by significant decreases in SOD, catalase, GPx, and GR activity in both pulmonary and splenic tissue. The enzymatic system was accompanied by a severe depletion of GSH. Conversely, GST activity exhibited a significant upsurge. Our findings lend support to previous research that reported the TiO_2_ NP significantly reduced the antioxidant enzymes^121,122^. This endogenous antioxidant system collapse is most likely caused by sustained high ROS levels^123^, due to TiO_2_ NPs tissue accumulation. SOD’s catalytic ability is functionally exhausted, causing a subsequent CAT and GPx failure. This’ redox collapse’ accounts for the dramatic drop in GSH levels as GR can’t keep up with the demand for GSH recycling, where GSH is either quickly consumed to neutralize ROS or conjugated by GST to detoxify free radicals derived from nanoparticles^124^.

Notably, co-administration of WGO significantly attenuated this toxicological systemic redox imbalance, where the nanoencapsulated WGO formulation demonstrated therapeutic potency, achieving no statistical difference from the healthy Control group for critical parameters such as CAT activity and GSH levels. In addition, attenuation of the GST increase may be explained by the preserved activity of the antioxidant system without the need to activate compensatory mechanisms. By providing a ‘molecular shield through its antioxidant content, WGO quenches free radicals before they can interact with the endogenous enzymes, effectively ‘sparing’ the enzymes and GSH from exhaustion. The continued superior efficacy of the nanoencapsulated WGO formulation is probably due to its optimized design and delivery characteristics. The effectiveness of WGO in protecting antioxidant enzyme activities has been previously demonstrated^125^.

#### Histopathological finding

To verify the observed biochemical and hematological results, a comprehensive histological evaluation was performed to characterize the morphological hallmarks of TiO_2_ NPs induced spleen and lung injury and the restorative efficacy of free and nanoencapsulated WGO.

##### Spleen histology

**Fig. 10:**
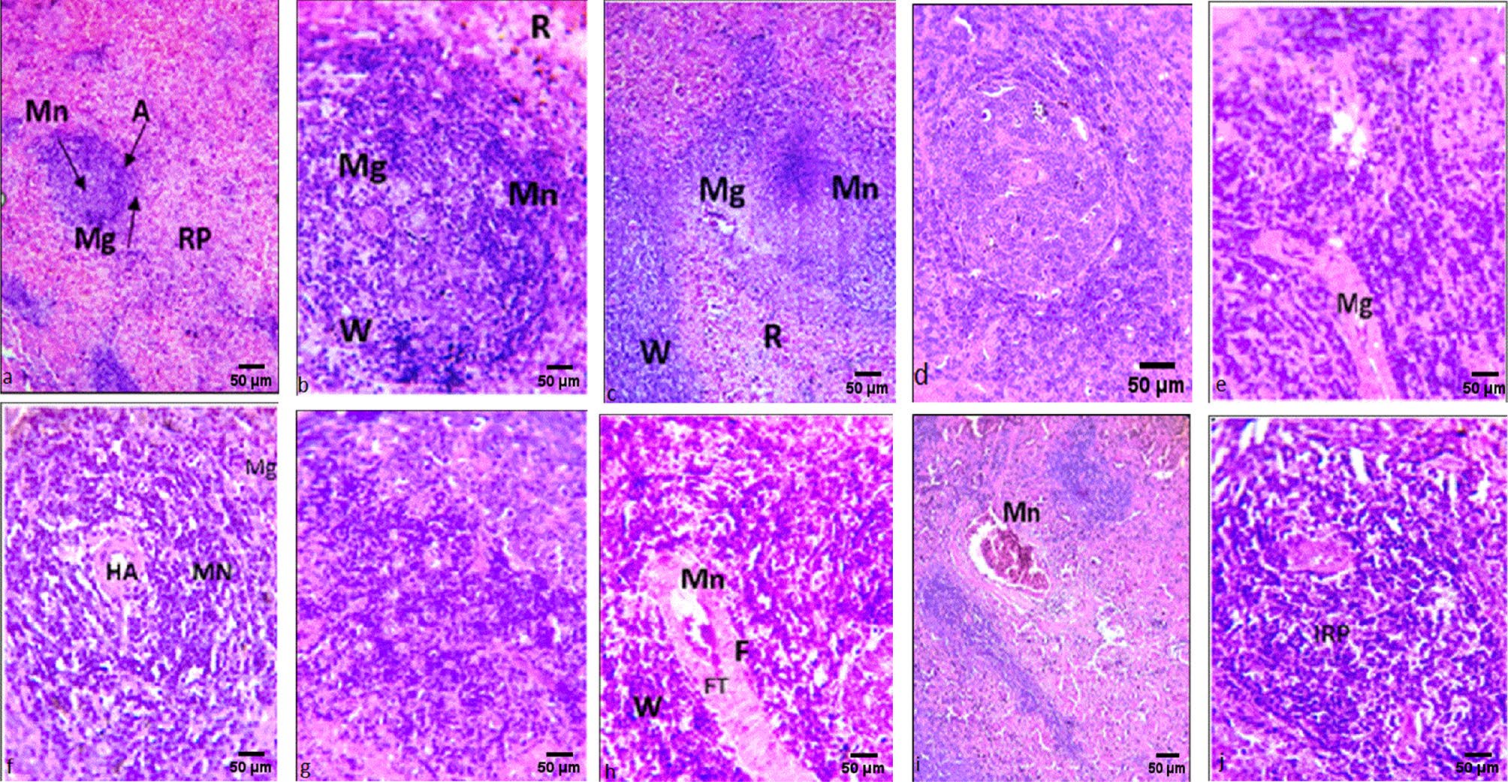
LM Micrograph rats’ spleen paraffin sections stained by H&E(X10), (**a**-**j**) Control group: untreated group, WGO, WGO NLC and placebo showing normal spleen tissue, typical splenic histology, central arteriol (A), mantle zone (Mn), marginal zone (Mg) of white pulp, normal red pulp (Rp). (e) rats’ spleen treated with TiO2(LD) showing moderate proliferation of cells of mantle zone (Mn), marginal zone (Mg) (disorganization of splenic compartments). (f) rats’ spleen treated with TiO2(LD) + WGO showing reduction of cells of mantle zone (Mn), marginal zone (Mg), rich white pulp (disorganization of splenic compartments). (g) rats’ spleen treated with TiO2(LD) + WGO NLC showing few infiltrations of red pulp and reduction of cells of Mantle zone, marginal zone, mild disorganization of splenic compartments. (h) rats’ spleen treated with TiO2(HD) showing disorganization of splenic compartments, infiltration and fibrotic activity of plasma cells of red pulp hypertrophic arteriole, proliferation of cells of mantle zone (Mn), marginal zone (Mg). (i) rats’ spleen treated with TiO2(HD) + WGO showing reduction of cells of mantle zone (Mn), marginal zone (Mg), and disorganization of splenic compartments. (j) rats’ spleen treated with TiO2(HD) + WGO NLC showing reduction of cells of mantle zone (Mn), marginal zone (Mg), mild disorganization of splenic compartments (H&E × 10,40).

The spleen, a central organ serving both immune and hematopoietic functions, is characterized by a highly organized architecture of white pulp (responsible for lymphoid function) and red pulp (responsible for hematopoietic filtration)^109^. As illustrated in Fig. 10**,** microscopic examination of splenic sections from the control, WGO, WGO NLC, and placebo groups exhibited normal histological features, with well-defined germinal centers, distinct mantle and marginal zones, and a healthy red pulp distribution. This is analogous to the biocompatibility results obtained from the hematological and biochemical examinations.

On the other hand, the low dose of TiO_2_ NPs caused a disorganized structure of the spleen compartments, characterized by hypertrophic mantle and marginal zone cell populations. This is indicative of the initiation of immunological reactions. In the high-dose group of TiO_2_ NPs, there were pathological changes characterized by hypertrophic arterioles, a high degree of tissue fibrosis, and tissue infiltration within the red pulp. This is indicative of a high degree of tissue disruption. This is analogous to the biphasic response of the leukocytes observed in hematological results. Initially, there is a hypertrophic response of the lymphoid tissue, which is followed by a reduction in the number of white blood cells. The tissue changes caused by the high dose of TiO_2_ NPs can be attributed to the reduced weight of the spleen. Biochemically, the experiments provided critical insights into the mechanisms of action of the nanoparticles. The results showed a critical depletion of antioxidant enzymes and a high level of oxidative stress markers. This is indicative of a systemic imbalance of biochemical reactions within the spleen. The results of the experiments are analogous to previous reports by^89,126,127^, which showed that TiO_2_ NPs act as immunotoxins within the spleen.

The preserving efficacy of free and nanoencapsulated co-administration of WGO was evident at all levels of analysis, showing significant preservation of splenic microenvironments. The histological examination indicated a significant reduction in splenic disorganization, cell proliferation, and fibrotic changes, with nanoencapsulated WGO showing greater maintenance of normal splenic architecture. The histopathological preservation was correlated with significant normalization of white blood cell counts, preservation of antioxidant activity, inhibition of lipid peroxidation, nitric oxide levels, and DNA damage, providing a comprehensive biochemical rationale for the preventative effects on splenic structure and systemic inflammatory response. The multifaceted approach used here has clearly established that TiO_2_ nanoparticles cause significant splenic immunotoxicity and systemic oxidative stress, which are significantly reduced by the prophylactic use of free and nanoencapsulated WGO. The results observed here are similar to those established by^128^, who showed that WGO has potential therapeutic applications in the treatment of acute toxoplasmosis infection, showing significant improvement in spleen histopathology.

##### Lung histology

**Fig. 11:**
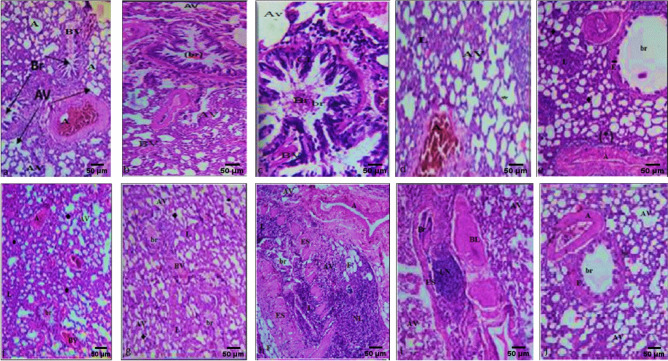
LM Micrograph rats’ lung paraffin sections stained by H&E(X400), (**a**-**d**) Control group: untreated group, WGO, WGO NLC and placebo showing normal architecture, normal bronchioles lumen and columnar epithelial cells, alveolar sacs surrounded by few leukocytes, non-filled alveoli, thin septa, and mild dilated blood vessels. (**e**) rats’ lung treated with TiO2(LD) showing thick alveolar septum (*) and more leukocyte infiltration (L), Congestion of blood vessels (BV), thick artery walls (A), and thick bronchial wall and squamous epithelial cells (E). (**f**) rats’ lung treated with TiO2(LD) + WGO showing area of recovering alveoli (AV) and decreased alveoli septum and decrease in leukocyte infiltration (L), decrease of congestion blood vessels and artery (A). (**g**) rats’ lung treated with TiO2(LD) + WGO NLC showing alveolar septum thickening and slight leukocyte. (**h**) rats’ lung treated TiO2(HD) showing marked degeneration of lung tissue, more infiltrating leukocytes from the follicular lymph node around alveolar sacs, presence of fatty alveolar sacs (F), increased and thick elastic fibers surrounding the degenerative bronchi fill with infiltrating leukocytes (L), have a narrow lumen (br), and elongated and thick-walled blood vessels (T). (**i**) rats’ lung treated with TiO2(HD) + WGO showing a marked improvement. The clear alveolar sacs (AV) decreased of leukocyte infiltration with reduced follicular node (LN) and absence of interstitial elastic fibers (ES), which reduced the bronchiolar wall. (**j**) rats’ lung treated with TiO2(HD) + WGO NLC showing improvement in many areas of alveolar sacs, the slightly thick septum with few leukocyte infiltrations, improved bronchiolar with clear lumen and cuboidal ciliated cells and normal congested artery (A) (H&E X40 Magn).

While the lungs are organs primarily recognized for gas exchange, their expansive microvascular network renders them a critical vascular target for systemically distributed toxicants. So, any lung pathology such as inflammation, fibrosis, oxidative stress, or toxic exposure can impair oxygen exchange and lead to compensatory or abnormal changes in hematological parameters, especially RBCs and Ht^129^. As shown in Fig. 11**,** pulmonary sections from the control, WGO, WGO-NLC, and placebo groups exhibited a well-preserved pulmonary histological architecture. These microscopic sections were characterized by clear bronchiolar lumens, thin alveolar septa, and patent alveolar sacs, confirming that the bioactive oil and the nanostructured lipid carrier are systemically biocompatible and do not induce inherent pulmonary irritation.

Due to their access through the systemic circulation, TiO_2_ NPs bypassed the upper respiratory tract’s initial defenses and localized accumulation of the nanoparticles within the lung parenchyma occurred through the alveolar-capillary interface. In the low-dose TiO_2_ NPs, the pulmonary tissue structure was characterized by a pronounced thickening of the alveolar septa with a significant leukocyte infiltrate, accompanied by vascular congestion and thickening of the arterial walls as well as bronchial walls. In the high-dose group of TiO_2_ NPs, there was a progression of pathological changes to severe degenerative states, with follicular lymphoid tissue surrounding the alveolar sacs, as well as fatty alveolar sacs. The presence of thickened elastic fibers as well as a constricted bronchiole lumen reflects a progression of pathological states towards fibrosis, which impairs ventilatory capacity. The mechanistic pathways underlying the observed histopathological changes can be attributed to a localized oxidative burst induced by TiO_2_ NPs, which catalyzes lipid peroxidation of pneumocyte membranes, leading to a breach in the structure of surfactant-producing type II pneumocytes as well as type I pneumocytes. The breach in type I pneumocytes is responsible for the inflammatory response, as well as septal edema, which is evident in the histopathological findings. The findings are consistent with established pulmonary toxicity profiles in line with previous studies in the literature^122,130^.

Prophylactic treatment with WGO significantly alleviated lung injury, with the nanoencapsulated WGO group showing remarkable preservation of alveolar structures, including patent alveolar sacs with clear lumens and normal cuboidal-shaped ciliated epithelial cells. This histological normalization correlates with the biochemical normalization of the endogenous antioxidant cascade reported in this study**,** in which WGO antioxidant constituents act as a systemic shield against membrane fragmentation. The superior efficacy of the nanoencapsulated WGO formulation can be explained by its optimized design and delivery characteristics. WGO maintains the functional integrity of the splenic-pulmonary axis and the gas exchange interface and prevents the systemic cascade of hematological and oxidative dysfunction. Moreover, the preventative capacity of WGO aligns with a previous study by^131^, which supports the potential effect of WGO as a prophylactic agent against nanoparticle-induced lung injury.

###### Immunohistochemical findings of lung tissue of male rats

**Fig. 12:**
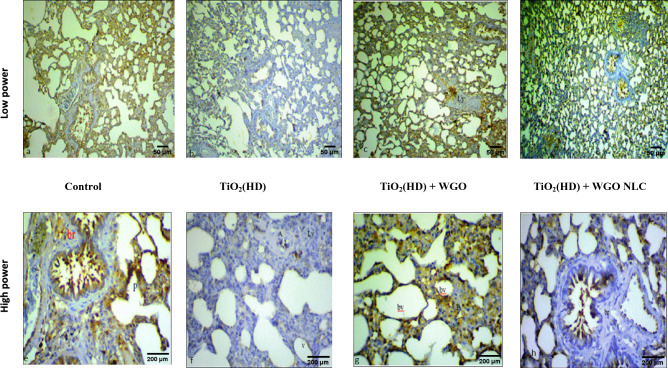
Photomicrograph of fixed formalin paraffin embedding FFPE immunostaining of EGFR protein in the control group (**a**, **e**), TiO_2_(HD) group (**b**, **f**), TiO_2_(HD) + WGO group (**c**, **g**) and TiO_2_(HD) + WGO NLC group (**d**, **h**) rats’ lung tissue. The (EGFR) expression indicated by dark brown stain labeling the tissue and the blue color indicated to the negative reaction. The semi quantitative evaluation of the immunohistochemically results of both primary antibodies done according to the intensity of stain (+ 1 weak, + 2 moderate, + 3 strong, + 4 intense).

Despite the lung’s significant relative susceptibility and vital role in gas exchange and its high susceptibility to injury, the observed elevated oxidative stress indicates the need for deeper molecular investigation. Consequently, immunohistochemical (IHC) mapping of the Epidermal Growth Factor Receptor (EGFR) was performed to evaluate how the high dose of TiO_2_ NPs induced oxidative burden disrupts the molecular integrity of the alveolar-capillary barrier. EGFR is a vital transmembrane receptor tyrosine kinase that plays a fundamental role in regulating cell growth, proliferation, and survival in the lung tissue. Its activation triggers intracellular signaling cascades that are critical for maintaining normal lung function^132^. The obtained photomicrographs of EGFR immunohistochemical staining in lung tissue from control rats and rats treated with TiO_2_ NPs (HD) alone or in combination with free or nanoencapsulated WGO are shown in Fig. 12**.** Lung tissue from the control group exhibited moderate EGFR immunoreactivity, visualized as brown DAB staining within the bronchiolar epithelial cells, pneumocytes, and endothelial cells lining the pulmonary vasculature. This staining pattern is consistent with the physiological role of EGFR in maintaining pulmonary tissue homeostasis.

Lung sections from rats exposed to the high dose of TiO_2_ NPs exhibited weak EGFR immunoreactivity, characterized by diffuse staining within the alveolar septa and bronchiolar epithelium. Immunoreactivity was markedly reduced in proliferating pneumocytes and was largely absent within lymphocytic aggregates. These findings are consistent with previous reports suggesting that TiO_2_ NP-induced oxidative stress and inflammation may interfere with EGFR-associated cellular signaling pathways and contribute to pulmonary tissue injury^17,133^. Prophylactic administration of free WGO attenuated the TiO_2_ NP-induced reduction in EGFR immunoreactivity, as evidenced by the presence of strong brown staining within bronchiolar epithelial cells and alveolar pneumocytes. This observation may be attributed, at least in part, to the antioxidant properties of WGO, which could help limit oxidative damage and preserve cellular integrity. Notably, the nanoencapsulated WGO group exhibited intense EGFR immunoreactivity than both the TiO_2_ NP-treated group and the group receiving free WGO. This enhanced staining was accompanied by improved preservation of lung architecture and reduced histopathological alterations. The increased EGFR immunoreactivity may suggest a more favorable environment for tissue maintenance and repair.

## Conclusion

The present study demonstrates that systemic exposure to TiO_2_ NPs induces dose-dependent oxidative stress and genomic instability, accompanied by structural and functional alterations in the lung and spleen, reflected by disruption of the alveolar–capillary barrier and alterations in tissue architecture. These changes were accompanied by hematological disturbances and consequent decline of erythrocytic lineage, suggesting systemic physiological impact. Prophylactic administration of free or nanoencapsulated WGO statistically maintained levels of biochemical, hematological, and histological parameters comparable to the control group and triggered compensatory EGFR upregulation, which is significant and primed the pulmonary microenvironment for an enhanced regenerative response and maintenance of structural integrity, even under high TiO_2_ NPs burden, with the superior efficacy of the nanoencapsulated form. The efficacy of WGO is noteworthy and has tremendous potential for use as a high-potency prophylactic agent for maintaining the functional vitality of splenic-pulmonary axis integrity. The scientific basis for using WGO as a biocompatible nano-delivery system is robust and has tremendous application for mitigating the increasing risks of TiO_2_ NPs toxicity and its impact on human health.

### Limitation and future work

The superior protective efficacy of WGO-NLC observed in the present study suggests that nanoencapsulation may enhance the bioavailability and tissue delivery of wheat germ oil bioactive constituents, thereby improving their antioxidant and cytoprotective activities. These findings support the potential translational value of WGO-NLC as a promising nutraceutical or adjunctive therapeutic strategy for mitigating nanoparticle-induced organ toxicity. Nevertheless, further investigations involving thermal characterization DCS, dose–response assessment, pharmacokinetic profiling, including temporal distribution kinetics, metabolite identification, and LC–MS/MS-based analysis, long-term safety evaluation, further studies investigating pH-dependent behavior and accelerated stability under different storage conditions would be valuable for future formulation optimization and translational development, and clinical studies are required before clinical application can be considered. Additionally, assessing inflammatory mediators and immunomodulatory markers such as TNF-α, IFN-γ, and NF-κB could provide additional mechanistic^134^, insight into the biological effects of TiO_2_ NPs-induced toxicity and the protective effects of WGO-NLC. Moreover, cellular cytotoxicity assessment^135^ using MTT or Trypan Blue assays would provide additional information regarding the biocompatibility and safety profile of the investigated formulation^136^.

## Supplementary Information

Supplementary Information.

## Data Availability

Data available upon request.

## References

[1] Anandalakshmi, K., Venugobal, J. & Ramasamy, V. Characterization of silver nanoparticles by green synthesis method using Pedalium murex leaf extract and their antibacterial activity. *Appl. Nanosci.* **6**(3), 399–408 (2016).

[2] Zaripova, O., Abdullaeva, M., To’raev, U., Rakhimova, G., Kadirova, L., Rajabova, Z., Temirova, D., Habibullaev, T., Yusupova, M., & Tursunova, G. (2025). *Long-term exposure to food colorants E171 and E173 leads to the accumulation of titanium and aluminum in the brain.* Paper presented at the BIO Web of Conferences.

[3] Thy, L. T. M., Dat, N. T. & Dat, N. M. Review on The Application of inorganic UV filters in Sunscreens: Mechanisms, Evaluation Methods, Toxicity, and Safety Enhancements. *Results Surfaces Interfaces* **20**, 100580 (2025).

[4] Sallam, M. F. et al. Improvement of the antioxidant activity of thyme essential oil against biosynthesized titanium dioxide nanoparticles-induced oxidative stress, DNA damage, and disturbances in gene expression in vivo. *J. Trace Elem. Med. Biol.* **73**, 127024 (2022). 35753172 10.1016/j.jtemb.2022.127024

[5] Jamkhande, P. G., Ghule, N. W., Bamer, A. H. & Kalaskar, M. G. Metal nanoparticles synthesis: An overview on methods of preparation, advantages and disadvantages, and applications. *J. Drug Deliv. Sci. Technol.* **53**, 101174 (2019).

[6] Boutillier, S., Fourmentin, S. & Laperche, B. History of titanium dioxide regulation as a food additive: a review. *Env. Chem. Letters* **20**, 1–17 (2022).

[7] Sallam, M. F. et al. Assessment of the oxidative damage and genotoxicity of titanium dioxide nanoparticles and exploring the protective role of holy basil oil nanoemulsions in rats. *Biol. Trace Elem. Res.* **201**(3), 1301–1316 (2023). 35416606 10.1007/s12011-022-03228-0 PMC9898350

[8] Lehotska Mikusova, M. et al. Titanium dioxide nanoparticles modulate systemic immune response and increase levels of reduced glutathione in mice after seven-week inhalation. *Nanomaterials (Basel, Switzerland)* **13**(4), 767 (2023). 36839135 10.3390/nano13040767 PMC9964099

[9] Wang, J. & Fan, Y. Lung injury induced by TiO2 nanoparticles depends on their structural features: size, shape, crystal phases, and surface coating. *Int. J. Mol. Sci.* **15**(12), 22258–22278 (2014). 25479073 10.3390/ijms151222258 PMC4284706

[10] Chen, Z. et al. Tissue-specific oxidative stress and element distribution after oral exposure to titanium dioxide nanoparticles in rats. *Nanoscale* **12**(38), 20033–20046 (2020). 32996981 10.1039/d0nr05591c

[11] Disdier, C. et al. Tissue biodistribution of intravenously administrated titanium dioxide nanoparticles revealed blood-brain barrier clearance and brain inflammation in rat. *Part. Fibre Toxicol.* **12**(1), 27 (2015). 26337446 10.1186/s12989-015-0102-8 PMC4559366

[12] Sheng, L. et al. Nano-sized titanium dioxide-induced splenic toxicity: A biological pathway explored using microarray technology. *J. Hazard. Mater.* **278**, 180–188 (2014). 24968254 10.1016/j.jhazmat.2014.06.005

[13] Cedervall, T. et al. Understanding the nanoparticle–protein corona using methods to quantify exchange rates and affinities of proteins for nanoparticles. *Proc. Natl. Acad. Sci. U. S. A.* **104**(7), 2050–2055 (2007). 17267609 10.1073/pnas.0608582104 PMC1892985

[14] Orrico, F. et al. Oxidative stress in healthy and pathological red blood cells. *Biomolecules* **13**(8), 1262 (2023). 37627327 10.3390/biom13081262 PMC10452114

[15] Sargazi, S. et al. Application of titanium dioxide nanoparticles in photothermal and photodynamic therapy of cancer: An updated and comprehensive review. *J. Drug Delivery Sci. Tech.* **75**, 103605 (2022).

[16] You, D. G. et al. ROS-generating TiO2 nanoparticles for non-invasive sonodynamic therapy of cancer. *Sci. Rep.* **6**(1), 23200 (2016). 26996446 10.1038/srep23200 PMC4800401

[17] Moon, C. et al. Pulmonary inflammation after intraperitoneal administration of ultrafine titanium dioxide (TiO2) at rest or in lungs primed with lipopolysaccharide. *J. Toxicol. Environ. Health A* **73**(5–6), 396–409 (2010). 20155581 10.1080/15287390903486543

[18] Shakeel, M. et al. Toxicity of nano-titanium dioxide (TiO2-NP) through various routes of exposure: A review. *Biol. Trace Elem. Res.* **172**(1), 1–36 (2016). 26554951 10.1007/s12011-015-0550-x

[19] Ghafoor, K. Nutritional composition, extraction, and utilization of wheat germ oil: A review. *Eur. J. Lipid Sci. Technol.* **119**(7), 1600160 (2017).

[20] El-Bana, M. A. et al. Formulation of wheat germ oil based on nanoemulsions to mitigate cisplatin’s nephrotoxic effects. *Prostaglandins Other Lipid Mediat.* **158**, 106603 (2022). 34852296 10.1016/j.prostaglandins.2021.106603

[21] Dey, S. et al. Design, synthesis and therapeutic exploration of nano-curcumin targeting the synergistic interactions with p53 and PARP-1 proteins in preventing food-additive induced genotoxicity and diabetic complications. *Colloids Surf. A Physicochem. Eng. Asp.* **710**, 136230 (2025).

[22] Apostolou, M., Assi, S., Fatokun, A. A. & Khan, I. The effects of solid and liquid lipids on the physicochemical properties of nanostructured lipid carriers. *J. Pharm. Sci.* **110**(8), 2859–2872 (2021). 33901564 10.1016/j.xphs.2021.04.012

[23] Bashiri, S., Ghanbarzadeh, B., Ayaseh, A., Dehghannya, J. & Ehsani, A. Preparation and characterization of chitosan-coated nanostructured lipid carriers (CH-NLC) containing cinnamon essential oil for enriching milk and anti-oxidant activity. *Lwt* **119**, 108836 (2020).

[24] Souto, E. B., Doktorovova, S., Zielinska, A. & Silva, A. M. Key production parameters for the development of solid lipid nanoparticles by high shear homogenization. *Pharm. Dev. Technol.* **24**(9), 1181–1185 (2019). 31354002 10.1080/10837450.2019.1647235

[25] Mura, P., Maestrelli, F., Cirri, M. & Mennini, N. Multiple roles of chitosan in mucosal drug delivery: An updated review. *Mar. Drugs* **20**(5), 335 (2022). 35621986 10.3390/md20050335 PMC9146108

[26] Hassan, D. M., El-Kamel, A. H., Allam, E. A., Bakr, B. A. & Ashour, A. A. Chitosan-coated nanostructured lipid carriers for effective brain delivery of Tanshinone IIA in Parkinson’s disease: Interplay between nuclear factor-kappa β and cathepsin B. *Drug Deliv. Transl. Res.* **14**(2), 400–417 (2024). 37598133 10.1007/s13346-023-01407-7 PMC10761445

[27] Tarrés, Q. et al. Dynamic light scattering plus scanning electron microscopy: Usefulness and limitations of a simplified estimation of nanocellulose dimensions. *Nanomaterials* **12**(23), 4288 (2022). 36500912 10.3390/nano12234288 PMC9739265

[28] Shi, F. et al. Preparation and characterization of solid lipid nanoparticles loaded with frankincense and myrrh oil. *Int. J. Nanomedicine*10.2147/IJN.S30085 (2012). 22619540 10.2147/IJN.S30085 PMC3356207

[29] Li, H. et al. Enhancement of gastrointestinal absorption of quercetin by solid lipid nanoparticles. *J. Control. Release* **133**(3), 238–244 (2009). 18951932 10.1016/j.jconrel.2008.10.002

[30] Jayari, A., Donsì, F., Ferrari, G. & Maaroufi, A. Nanoencapsulation of thyme essential oils: Formulation, characterization, storage stability, and biological activity. *Foods* **11**(13), 1858 (2022). 35804672 10.3390/foods11131858 PMC9265609

[31] Braca, A. et al. Antioxidant principles from *Bauhinia tarapotensis*. *J. Nat. Prod.* **64**(7), 892–895 (2001). 11473417 10.1021/np0100845

[32] Oyaizu, M. Studies on products of browning reaction antioxidative activities of products of browning reaction prepared from glucosamine. *Japanese J. Nutrition Dietetics* **44**(6), 307–315 (1986).

[33] Abdel-Halim, K. Y., Osman, S. R., Abuzeid, M. A., El-Danasoury, H. T. & Khozimy, A. M. Apoptotic and histopathological defects enhanced by titanium dioxide nanoparticles in male mice after short-term exposure. *Toxicol. Rep.* **9**, 1331–1346 (2022). 36518392 10.1016/j.toxrep.2022.06.003 PMC9743451

[34] Salman, A. S. et al. Matlodextrin-cinnamon essential oil nanoformulation as a potent protective against titanium nanoparticles-induced oxidative stress, genotoxicity, and reproductive disturbances in male mice. *Environ. Sci. Pollut. Res.* **28**, 39035–39051 (2021). 10.1007/s11356-021-13518-0 33745051

[35] Younes, N. R. B. et al. Subacute toxicity of titanium dioxide (TiO_2_) nanoparticles in male rats: Emotional behavior and pathophysiological examination. *Environ. Sci. Pollut. Res.* **22**, 8728–8737 (2015). 10.1007/s11356-014-4002-5 25572266

[36] Hussein, S. A., Abdel-Aal, S. & Elghwab, A. Biochemical role of wheat germ oil on biomarkers of oxidative stress and inflammatory response in a rat model of endotoxemia. *Benha Vet Med J* **27**(2), 157–167 (2014).

[37] Festing, M. F. & Altman, D. G. Guidelines for the design and statistical analysis of experiments using laboratory animals. *ILAR J.* **43**(4), 244–258 (2002). 12391400 10.1093/ilar.43.4.244

[38] Wintrobe, M. M. *Wintrobe’s clinical hematology* Vol. Vol. 1 (Lippincott Williams & Wilkins, 2009).

[39] Montgomery, H. The determination of nitrite in water. *Analyst* **1**, 123–130 (1971).

[40] Tappel, A. & Zalkin, H. Inhibition of lipide peroxidation in mitochondria by vitamin E. *Arch. Biochem. Biophys.* **80**(2), 333–336 (1959).

[41] DM, G. (1983). Assay of glutathione reductase. *Methods in enzymatic analysis*.

[42] Habig, W. H., Pabst, M. J. & Jakoby, W. B. Glutathione S-transferases: the first enzymatic step in mercapturic acid formation. *J. Biol. Chem.* **249**(22), 7130–7139 (1974). 4436300

[43] Nishikimi, M., Rao, N. A. & Yagi, K. The occurrence of superoxide anion in the reaction of reduced phenazine methosulfate and molecular oxygen. *Biochem. Biophys. Res. Commun.* **46**(2), 849–854 (1972). 4400444 10.1016/s0006-291x(72)80218-3

[44] Paglia, D. E. & Valentine, W. N. Studies on the quantitative and qualitative characterization of erythrocyte glutathione peroxidase. *J. Lab. Clin. Med.* **70**(1), 158–169 (1967). 6066618

[45] Sinha, A. K. Colorimetric assay of catalase. *Anal. Biochem.* **47**(2), 389–394 (1972). 4556490 10.1016/0003-2697(72)90132-7

[46] Ellman, M. A spectrophotometric method for determination of reduced glutathione in tissues. *Anal Biochem* **74**(1), 214–226 (1959). 10.1016/0003-2697(76)90326-2 962076

[47] Wu, B. et al. T cell deficiency leads to liver carcinogenesis in azoxymethane-treated rats. *Exp. Biol. Med.* **231**(1), 91–98 (2006). 10.1177/153537020623100111 16380649

[48] Eilola, K. & Perämäki, P. Microwave heated vapor-phase digestion method for biological sample materials. *Fresenius J. Anal. Chem.* **369**(1), 107–112 (2001). 11210223 10.1007/s002160000611

[49] Suvarna, K. S., Layton, C. & Bancroft, J. D. *Bancroft’s theory and practice of histological techniques E-Book* (Elsevier health sciences, 2018).

[50] Salguero, F., Mekonnen, T., Ruiz-Villamor, E., Sánchez-Cordón, P. & Gómez-Villamandos, J. Detection of monokines in paraffin-embedded tissues of pigs using polyclonal antibodies. *Vet. Res.* **32**(6), 601–609 (2001). 11777010 10.1051/vetres:2001103

[51] Norušis, M. J. *SPSS 14.0 guide to data analysis* (Prentice Hall, 2006).

[52] Emam, K. K., Abdel Fattah, M. E., El Rayes, S. M., Hebishy, M. A. & Dessouki, A. A. Assessment of wheat germ oil role in the prevention of induced breast cancer in rats. *ACS Omega* **7**(16), 13942–13952 (2022). 35559156 10.1021/acsomega.2c00434 PMC9089347

[53] Yaseen, A. A. et al. Potential protection from Alzheimer’s disease by wheat germ and rice bran nano-form in rat model. *J. Appl. Pharm. Sci.* **9**(1), 067–076 (2019).

[54] Zargar, S., Wani, T. A. & Rizwan Ahamad, S. An insight into wheat germ oil nutrition, identification of its bioactive constituents and computer-aided multidimensional data analysis of its potential anti-inflammatory effect via molecular connections. *Life* **13**(2), 526 (2023). 36836883 10.3390/life13020526 PMC9960255

[55] Dachev, M., Bryndová, J., Jakubek, M., Moučka, Z. & Urban, M. The effects of conjugated linoleic acids on cancer. *Processes* **9**(3), 454 (2021).

[56] Naghshi, S. et al. Dietary intake and biomarkers of alpha linolenic acid and risk of all cause, cardiovascular, and cancer mortality: systematic review and dose-response meta-analysis of cohort studies. *BMJ*10.1136/bmj.n2213 (2021). 34645650 10.1136/bmj.n2213 PMC8513503

[57] Hu, W., Fitzgerald, M., Topp, B., Alam, M. & O’Hare, T. J. A review of biological functions, health benefits, and possible de novo biosynthetic pathway of palmitoleic acid in macadamia nuts. *Journal of Functional Foods* **62**, 103520 (2019).

[58] Wang, Y. et al. Omega-9 monounsaturated fatty acids: A review of current scientific evidence of sources, metabolism, benefits, recommended intake, and edible safety. *Crit. Rev. Food Sci. Nutr.* **65**(10), 1857–1877 (2025). 38343184 10.1080/10408398.2024.2313181

[59] Zhu, S. et al. Palmitic acid inhibits prostate cancer cell proliferation and metastasis by suppressing the PI3K/Akt pathway. *Life Sci.* **286**, 120046 (2021). 34653428 10.1016/j.lfs.2021.120046

[60] Sakthivel, R., Sheeja Malar, D., Archunan, G. & Pandima Devi, K. Phytol ameliorated benzo (a) pyrene induced lung carcinogenesis in Swiss albino mice via inhibition of oxidative stress and apoptosis. *Environ. Toxicol.* **34**(4), 355–363 (2019). 30520250 10.1002/tox.22690

[61] Shalabi, A. & Eskander, D. M. Isolation of secondary metabolites from marine Streptomyces sparsus ASD203 and evaluation its bioactivity. *Egypt. J. Chem.* **65**(3), 539–547 (2022).

[62] Gasa-Falcon, A., Odriozola-Serrano, I., Oms-Oliu, G. & Martín-Belloso, O. Nanostructured lipid-based delivery systems as a strategy to increase functionality of bioactive compounds. *Foods* **9**(3), 325 (2020). 32168809 10.3390/foods9030325 PMC7143550

[63] Diniz, F. et al. dPolymorphism, crystallinity and hydrophilic-lipophilic balance (HLB) of cetearyl alcohol and cetyl alcohol as raw materials for solid lipid nanoparticles (SLN). *Asp. Nanotechnol* **1**, 52–60 (2018).

[64] Elmowafy, M. et al. Fatty alcohol containing nanostructured lipid carrier (NLC); Strategy to fade away progesterone oral delivery drawbacks. *J. Drug Deliv. Sci. Technol* **45**, 230 (2018).

[65] Nadzir, M. M., Fen, T. W., Mohamed, A. R. & Hisham, S. F. Size and stability of curcumin niosomes from combinations of Tween 80 and Span 80. *Sains Malaysiana* **46**(12), 2455–2460 (2017).

[66] Honary, S. & Zahir, F. Effect of zeta potential on the properties of nano-drug delivery systems-a review (Part 2). *Trop. J. Pharm. Res.* **12**(2), 265–273 (2013).

[67] Zeng, L., Xin, X. & Zhang, Y. Development and characterization of promising Cremophor EL-stabilized o/w nanoemulsions containing short-chain alcohols as a cosurfactant. *RSC Adv.* **7**(32), 19815–19827 (2017).

[68] Ajiboye, A. L., Nandi, U., Galli, M. & Trivedi, V. Olanzapine loaded nanostructured lipid carriers via high shear homogenization and ultrasonication. *Sci. Pharm.* **89**(2), 25 (2021).

[69] Aibani, N., Rai, R., Patel, P., Cuddihy, G. & Wasan, E. K. Chitosan nanoparticles at the biological interface: Implications for drug delivery. *Pharmaceutics* **13**(10), 1686 (2021). 34683979 10.3390/pharmaceutics13101686 PMC8540112

[70] Yue, Z.-G. et al. Surface charge affects cellular uptake and intracellular trafficking of chitosan-based nanoparticles. *Biomacromol* **12**(7), 2440–2446 (2011). 10.1021/bm101482r 21657799

[71] Mirtalebi, M., Rajaei, A., Bahmaei, M. & Yari Khosroushahi, A. Storage stability of wheat germ oil encapsulated within nanostructured lipid carriers. *Journal of Nanostructures* **10**(2), 268–278 (2020).

[72] Rana, A. et al. An investigation of antimicrobial activity for plant pathogens by green-synthesized silver nanoparticles using Azadirachta indica and Mangifera indica. *Physchem* **3**(1), 125–146 (2023).

[73] Nagar, N. & Devra, V. A kinetic study on the degradation and biodegradability of silver nanoparticles catalyzed methyl orange and textile effluents. *Heliyon* **5**(3), e01356 (2019). 30957040 10.1016/j.heliyon.2019.e01356 PMC6431746

[74] Guillén, M. D. & Cabo, N. Infrared spectroscopy in the study of edible oils and fats. *J. Sci. Food Agric.* **75**(1), 1–11 (1997).

[75] Rohman, A. & Man, Y. C. Application of Fourier transform infrared spectroscopy for authentication of functional food oils. *Appl. Spectrosc. Rev.* **47**(1), 1–13 (2012).

[76] Paśko, P. et al. Molecular profiling and FTIR characterization of wheat germ oil, supported by the screening of its anti-inflammatory and cytotoxic properties. *Biomolecules* **15**(4), 464 (2025). 40305174 10.3390/biom15040464 PMC12025205

[77] Brugnerotto, J. et al. An infrared investigation in relation with chitin and chitosan characterization. *Polymer* **42**(8), 3569–3580 (2001).

[78] Kasaai, M. R. A review of several reported procedures to determine the degree of N-acetylation for chitin and chitosan using infrared spectroscopy. *Carbohyd. Polym.* **71**(4), 497–508 (2008).

[79] Ghagane, S. C. et al. In vitro antioxidant and anticancer activity of *Leea indica* leaf extracts on human prostate cancer cell lines. *Integr. Med. Res.* **6**(1), 79–87 (2017). 28462147 10.1016/j.imr.2017.01.004 PMC5395687

[80] Arslan, D., Demir, M. K., Acar, A. & Arslan, F. N. Investigation of wheat germ and oil characteristics with regard to different stabilization techniques. *Food Technol. Biotechnology.* **58**(3), 348–355 (2020). 10.17113/ftb.58.03.20.6638 PMC7709457 33281490

[81] Liaqat, H. et al. Antioxidant effect of wheat germ extracts and their antilipidemic effect in palmitic acid-induced steatosis in HepG2 and 3T3-L1 cells. *Foods* **10**(5), 1061 (2021). 34065831 10.3390/foods10051061 PMC8151358

[82] Jumina, J. et al. Development of C-arylcalix [4] resorcinarenes and C-arylcalix [4] pyrogallolarenes as antioxidant and UV-B protector. *Indones. J. Chem.* **19**(2), 273–284 (2019).

[83] Tian, S., Meng, F., Du, K. & Chen, Y. Biological activity evaluation and identification of different molecular weight peptides from wheat germ albumin. *Lwt* **189**, 115556 (2023).

[84] Zhu, K.-X., Lian, C.-X., Guo, X.-N., Peng, W. & Zhou, H.-M. Antioxidant activities and total phenolic contents of various extracts from defatted wheat germ. *Food Chem.* **126**(3), 1122–1126 (2011).

[85] Wu, F. et al. Antioxidant capacities of heat-treated wheat germ and extruded compounded bran. *Cereal Chem.* **99**(3), 582–592 (2022).

[86] Nagib, R. Protective effect of wheat germ powder and oil on metabolic disturbances in rats. *J. Food Dairy Sci.* **3**(3), 21–27 (2018).

[87] Jia, F., Sun, Z., Yan, X., Zhou, B. & Wang, J. Effect of pubertal nano-TiO 2 exposure on testosterone synthesis and spermatogenesis in mice. *Arch. Toxicol.* **88**, 781–788 (2014). 24241477 10.1007/s00204-013-1167-5

[88] Jia, X., Wang, S., Zhou, L. & Sun, L. The potential liver, brain, and embryo toxicity of titanium dioxide nanoparticles on mice. *Nanoscale Res. Lett.* **12**(1), 1–14 (2017). 28774157 10.1186/s11671-017-2242-2 PMC5540742

[89] Afshari-Kaveh, M., Abbasalipourkabir, R., Nourian, A. & Ziamajidi, N. The protective effects of vitamins a and e on titanium dioxide nanoparticles (nTiO2)-induced oxidative stress in the spleen tissues of male Wistar rats. *Biol. Trace Elem. Res.* **199**, 3677–3687 (2021). 33210191 10.1007/s12011-020-02487-z

[90] Lyu, S. et al. Titanium as a beneficial element for crop production. *Front. Plant Sci.* **8**, 597 (2017). 28487709 10.3389/fpls.2017.00597 PMC5404504

[91] Tighe-Neira, R. et al. Physiological and agronomical traits effects of titanium dioxide nanoparticles in seedlings of *Solanum lycopersicum* L. *BMC Plant Biol.* **24**(1), 146. (2024). 38413850 10.1186/s12870-024-04763-9 PMC10900795

[92] Heringa, M. et al. Detection of titanium particles in human liver and spleen and possible health implications. *Part. Fibre Toxicol.* **15**, 1–9 (2018). 29642936 10.1186/s12989-018-0251-7 PMC5896156

[93] Wani, M. R., Maheshwari, N. & Shadab, G. Eugenol attenuates TiO_2_ nanoparticles-induced oxidative damage, biochemical toxicity and DNA damage in Wistar rats: An in vivo study. *Environ. Sci. Pollut. Res. Int.* **28**, 22664–22678 (2021). 33420693 10.1007/s11356-020-12139-3

[94] Kreyling, W. G. et al. Quantitative biokinetics of titanium dioxide nanoparticles after oral application in rats: Part 2. *Nanotoxicology* **11**(4), 443–453 (2017). 28290734 10.1080/17435390.2017.1306893

[95] Sang, X. et al. The chronic spleen injury of mice following long-term exposure to titanium dioxide nanoparticles. *Journal of biomedical materials research. Part A* **100**(4), 894–902 (2012). 22275130 10.1002/jbm.a.34024

[96] Boughediri, K., Ouazouaz, M., Triki, R. & Henchiri, C. The therapeutic potential of saharian monovarietal virgin olive oil (olea europea l.) on hematological, histological, and antioxidant status in titanium dioxide nanoparticles-induced oxidative stress in rats. *Comparative Clinical Pathol.* **33**(3), 411–423 (2024).

[97] Grissa, I. et al. Anemia and genotoxicity induced by sub-chronic intragastric treatment of rats with titanium dioxide nanoparticles. *Mutat. Res. Genet. Toxicol. Environ. Mutagen.* **794**, 25–31 (2015). 26653980 10.1016/j.mrgentox.2015.09.005

[98] Abdou, H. M., Mohamed, N. A., El Mekkawy, D. A. & EL-Hengary, S. B.,. Vitamin E and/or wheat germ oil supplementation ameliorate oxidative stress induced by cadmium chloride in pregnant rats and their fetuses. *Jordan J. Biological Sci.* **10**(1), 39–48 (2017).

[99] Oruch, R. & Pryme, I. F. The biological significance of vitamin A in humans: A review of nutritional aspects and clinical considerations. *Science Jet* **1**(19), 1–13 (2012).

[100] Saleh, N. Evaluation of Protective Effect of Wheat Germ on Chlorpyrifos Induced Toxicity in Rats. *Int. J. Adv. Res.* **5**(12), 991–1002 (2017).

[101] El-Nashar, H. A. et al. Neuroprotective effect of artichoke-based nanoformulation in sporadic Alzheimer’s disease mouse model: Focus on antioxidant, anti-inflammatory, and amyloidogenic pathways. *Pharmaceuticals* **15**(10), 1202 (2022). 36297313 10.3390/ph15101202 PMC9610800

[102] Elkholy, N. E., Sultan, A. A., Abu-Risha, S. E. & El Maghraby, G. M. Chitosan coated lipid carriers as nanoplatform for repurposed anti-breast cancer activity of niclosamide. *J. Drug Deliv. Sci. Technol.* **93**, 105414 (2024).

[103] Samadder, A. et al. Possible signaling cascades involved in attenuation of alloxan-induced oxidative stress and hyperglycemia in mice by ethanolic extract of *Syzygium jambolanum*: Drug-DNA interaction with calf thymus DNA as target. *Eur. J. Pharm. Sci.* **44**(3), 207–217 (2011). 21839831 10.1016/j.ejps.2011.07.012

[104] An, H. et al. Oxidative damage induced by nano-titanium dioxide in rats and mice: A systematic review and meta-analysis. *Biol. Trace Elem. Res.* **194**, 184–202 (2020). 31342340 10.1007/s12011-019-01761-z

[105] Musial, J., Krakowiak, R., Mlynarczyk, D. T., Goslinski, T. & Stanisz, B. J. Titanium dioxide nanoparticles in food and personal care products—What do we know about their safety?. *Nanomaterials (Basel, Switzerland)* **10**(6), 1110 (2020). 32512703 10.3390/nano10061110 PMC7353154

[106] Rizk, M. Z. et al. Toxicity of titanium dioxide nanoparticles: Effect of dose and time on biochemical disturbance, oxidative stress and genotoxicity in mice. *Biomed. Pharmacother.* **90**, 466–472 (2017). 28391168 10.1016/j.biopha.2017.03.089

[107] Abuzaied, H., Bashir, D. W., Rashad, E., Rashad, M. M. & El-Habback, H. Ginseng Extract can alleviate The Induced-renal Toxicity of Titanium Dioxide Nanoparticles in a Rat Model. *J. Appl. Veterinary Sci.* **9**(2), 1–17 (2024).

[108] Eid, A. et al. Hesperidin attenuates titanium dioxide nanoparticle-induced neurotoxicity in rats by regulating Nrf-2/TNF-α signaling pathway, the suppression of oxidative stress, and inflammation. *ACS Omega* **8**(40), 37584–37591 (2023). 37841165 10.1021/acsomega.3c06198 PMC10568688

[109] Ibrahim, R., Elkady, M. & Hassanein, A. Effect of some antioxidants on rats treated with Titanium dioxide nanoparticles. *Egyptian Journal of Food Science* **47**(1), 91–103 (2019).

[110] Ganz, T. & Nemeth, E. Iron homeostasis in host defence and inflammation. *Nat. Rev. Immunol.* **15**(8), 500–510 (2015). 26160612 10.1038/nri3863 PMC4801113

[111] Weiss, G. & Goodnough, L. T. Anemia of chronic disease. *N. Engl. J. Med.* **352**(10), 1011–1023 (2005). 15758012 10.1056/NEJMra041809

[112] Chen, T., Yan, J. & Li, Y. Genotoxicity of titanium dioxide nanoparticles. *J. Food Drug Anal.* **22**(1), 95–104 (2014). 24673907 10.1016/j.jfda.2014.01.008 PMC9359145

[113] Han, B. et al. TiO2 nanoparticles caused DNA damage in lung and extra-pulmonary organs through ROS-activated FOXO3a signaling pathway after intratracheal administration in rats. *Int. J. Nanomedicine* **15**, 6279–6294 (2020). 32904047 10.2147/IJN.S254969 PMC7449758

[114] Ze, Y. et al. Molecular mechanism of titanium dioxide nanoparticles-induced oxidative injury in the brain of mice. *Chemosphere* **92**(9), 1183–1189 (2013). 23466083 10.1016/j.chemosphere.2013.01.094

[115] Zhang, W. et al. Alleviative effect of lactoferrin interventions against the hepatotoxicity induced by titanium dioxide nanoparticles. *Biol. Trace Elem. Res.* **202**(2), 624–642 (2024). 37191759 10.1007/s12011-023-03702-3

[116] Bonetta, S. et al. DNA damage in workers exposed to pigment grade titanium dioxide (TiO2) and association with biomarkers of oxidative stress and inflammation. *Environ. Toxicol. Pharmacol.* **105**, 104328 (2024). 38013010 10.1016/j.etap.2023.104328

[117] Akool, E.-S. Molecular mechanisms of the protective role of wheat germ oil against oxidative stress–induced liver disease. In *Dietary interventions in liver disease* 233–238 (Elsevier, 2019).

[118] Liu, C. et al. The hypolipidemic and antioxidant activity of wheat germ and wheat germ protein in high-fat diet-induced rats. *Molecules* **27**(7), 2260 (2022). 35408659 10.3390/molecules27072260 PMC9000699

[119] Saleh, H. Modulatory effect of wheat germ oil on intestinal oxidative stress and DNA damage induced by carbon tetrachloride in Mice. *J. Appl. Pharm. Sci.* **6**(12), 067–074 (2016).

[120] Abdallah, A. & Bakry, S. Hepatoprotective and Antioxidant Activity of Wheat Germ Oil Against Nicotine Induced Oxidative Stress. *Advances in Biological Research* **8**(6), 289–297 (2014).

[121] Abdel-Halim, K., Osman, S., Abuzeid, M. & Khozimy, A. Hematological Changes and Oxidative Stress Induction of Titanium Dioxide Nanoparticles in Male Mice after Intraperitoneal Injection of Different Doses for 28 Days: Study of Organ’s Responsibility. *NanoWorld J* **7**(2), 22–32 (2021).

[122] Sun, Q. et al. Pulmotoxicological effects caused by long-term titanium dioxide nanoparticles exposure in mice. *J. Hazard. Mater.* **235**, 47–53 (2012). 22898172 10.1016/j.jhazmat.2012.05.072

[123] Dey, R. et al. Novel PLGA-encapsulated-nanopiperine promotes synergistic interaction of p53/PARP-1/Hsp90 axis to combat ALX-induced-hyperglycemia. *Sci. Rep.* **14**(1), 9483. (2024). 38664520 10.1038/s41598-024-60208-1 PMC11045756

[124] Amin, D. M., Abohashem, A. A., Amer, S. A., Ahmed, A. I. & Moustafa, A. A. Therapeutic assessment of Idebenone versus Titanium dioxide nanoparticles induced pulmonary injury in adult albino Rats: Experimental study. *J. Toxicol. Env. Health Sci.* **11**(6), 62–74 (2019).

[125] Elgendy, S. A. et al. Ameliorative impacts of wheat germ oil against ethanol-induced hepatic and renal dysfunction in rats: Involvement of anti-inflammatory, anti-apoptotic, and antioxidant signaling pathways. *Life (Basel, Switzerland)* **12**(10), 1671 (2022). 36295108 10.3390/life12101671 PMC9605469

[126] Ibrahim, R., Salem, M. Y., Helal, O. K. & Abd El-Monem, S. N. Effect of titanium dioxide nanoparticles on the spleen of adult male albino rats: Histological and immunohistochemical study. *Egyptian Journal of Histology* **41**(3), 311–328 (2018).

[127] Jaber, F. A. Quercetin mitigates oxidative stress, inflammation, apoptosis, and histopathological alterations induced by chronic titanium dioxide nanoparticle exposure in the rat spleen. *Microsc. Microanal.* **29**(5), 1718–1729 (2023). 37584520 10.1093/micmic/ozad081

[128] Barakat, A. M. et al. Parasitological, molecular, and histopathological investigation of the potential activity of propolis and wheat germ oil against acute toxoplasmosis in mice. *Pharmaceutics* **15**(2), 478 (2023). 36839800 10.3390/pharmaceutics15020478 PMC9967381

[129] Shi, H., Magaye, R., Castranova, V. & Zhao, J. Titanium dioxide nanoparticles: A review of current toxicological data. *Part. Fibre Toxicol.* **10**(1), 15 (2013). 23587290 10.1186/1743-8977-10-15 PMC3637140

[130] Doudi, M. & Setorki, M. Influence of titanium dioxide nanoparticles on oxidative stress and pulmonary dysfunction. *Zahedan Journal of Research in Medical Sciences* **17**(9), e1062 (2015).

[131] Barakat, A. M. et al. Wheat germ oil and propolis decrease parasite burden and restore marked histopathological changes in liver and lung in mice with chronic toxoplasmosis. *Animals : an open access journal from MDPI* **12**(22), 3069 (2022). 36428297 10.3390/ani12223069 PMC9686545

[132] Ramos-Vara, J. A. (2010). Principles and methods of immunohistochemistry. *Drug safety evaluation: Methods and protocols*, 83–96. 10.1007/978-1-60761-849-2_5 20972748

[133] Kim, H. et al. Titanium dioxide nanoparticles induce apoptosis by interfering with EGFR signaling in human breast cancer cells. *Environ. Res.* **175**, 117–123 (2019). 31112848 10.1016/j.envres.2019.05.001

[134] Kelkar, A. et al. Exploring the effects of zinc oxide nanoflakes synthesized from structurally diverse templates on cellular apoptosis and necrosis in *Candida albicans*. *Mater. Lett.* **409**, 140205 (2026).

[135] Samadder, A. et al. The potentized homeopathic drug, *Lycopodium clavatum* (5C and 15C) has anti-cancer effect on hela cells in vitro. *J. Acupunct. Meridian Stud.* **6**(4), 180–187 (2013). 23972240 10.1016/j.jams.2013.04.004

[136] Choudhary, S. et al. Engineering the microenvironment: advanced biomaterials for humanized in vitro immunotoxicology and carcinogenicity assessment. *Exploration of BioMat-X* **2**, 101351 (2025).

