# Supplementary material for: Comparative attenuation of TiO₂ nanoparticle dose-dependent toxicities in lung, spleen, and blood by nanoencapsulated wheat germ oil in male rats
Source: Sci Rep. 2026 Jul 8;16:21225. doi: 10.1038/s41598-026-59659-5 (PMC13346894; doi:10.1038/s41598-026-59659-5)
Supplement: Supplementary file 1 — Supplementary Information. [file 41598_2026_59659_MOESM1_ESM.docx]

1. **Materials and methods**

**Table 1: Gas Chromatography-Mass Spectroscopy Condition**

| Carrier gas: | Helium (average velocity 39 cm/s) |
| --- | --- |
| Flow rate | 0.7 mL/min |
| Run time | 43.67 min |
| The temperature program of the oven | Increased from 50 ºC for 1 min (6ºC/min) to 200ºC, increased from 200 ºC to 270 ºC for 5 min (6ºC/min). |
| Column type | A TG-5MS Zebron capillary column (length 30m× 0.25 mm ID, 0.25 µm film thickness; Thermo) |
| Injector temperature | 300ºC |
| Sample volume | 1 µL |
| Injection type | split |
| Split ratio | 20:1 |
| Electron impact ionization (EI) | 70 electron volts |
| Scanning range | 50-600 m/zat five scans per second |
| MS libraries | NIST database |
| Solvent type | Hexane |

- 1. **Determination of scavenging activity of wheat germ oil against 1, 1-Diphenyl-2-picrylhydrazyl DPPH**

This assay measures the free radical scavenging capacity of WGO against the stable free radical DPPH in the presence of an antioxidant that can donate an electron to DPPH. The purple color typical of the free DPPH radical decays, and the absorbance change at 517 nm was measured. This test provides information on a compound's ability to donate a hydrogen atom as the mechanism of antioxidant action.

**Reagents**

- DPPH (0.004% in methanol)
- Positive control: serial dilution of vitamin E (0.1 – 2.0 mg/mL in absolute methanol)
- Negative control: 100 μL DPPH in 100μl methanol
- Sample: serial dilution (0.1 – 2.0) in absolute methanol

**Procedure**

The assay mixture, contained in a total volume of 100 μL of prepared DPPH, was added to 100 μL of the oil or vitamin E as a positive control. The plate was shaken to ensure thorough mixing before being wrapped with aluminum foil and placed into the dark for 30 min at 25°C. Then the decrease in absorbance was measured at 517 nm using an Optima spectrophotometer. The test was carried out in triplicate.

**Calculation**

The radical scavenging activity was calculated from the following equation:

**Percentage of free radical scavenging activity (inhibition %) = [AC - AE) /AC] × 100**

Where:

AC: The mean of the absorbance of the negative control

AE: The mean of the absorbance of oil

From the inhibition curve, **IC50** was calculated (which represents the concentration of the sample required to reduce the initial DPPH concentration by 50%, and is a commonly used parameter to assess antiradical efficiency.

**Y= ax + c**

Y= 50

a= slope

X = concentration of oil to inhibit 50 % DPPH

C= intercept

- 1. **Ferric reducing antioxidant power capacity (FRAP)**

Reducing the capacity of WGO was monitored by ferric reducing antioxidant power capacity. Ferric ion (Fe+3) is generated by potassium ferric cyanide, reduced by antioxidants such as flavonoids and phenolic acids in oil, resulting in the formation of a blue ferrous complex.

**Reagents**

- Standard (positive control): Vitamin E and sample serial dilutions (0.01 - 0.2 mg/mL) were dissolved in absolute methanol.
- Phosphate buffer (0.2M, pH=6.6); 0.53 g disodium hydrogen phosphate and 0.93 g dihydrogen sodium phosphate were dissolved in 48.9 mL distilled H_2_O.
- Potassium ferricyanide (K₃ [Fe(CN)₆], 1%): 0.5 g potassium ferricyanide was dissolved in 50 ml distilled H2O.
- Trichloroacetic acid (TCA, 10%); 5 g trichloroacetic acid was dissolved in 50 mL distilled H_2_O.
- Ferric chloride (FeCl3, 0.1%); 0.05 g ferric chloride was dissolved in 50 mL distilled H_2_O.

**Procedure**

One mL of prepared phosphate buffer and 1ml potassium ferricyanide were added and mixed with an aliquot (1mL) of sample/ vitamin E for standard tubes (1,5, 10, 15, 20%). In sample blank/ blank tubes were prepared similarly, replacing potassium ferricyanide with ethanol/H_2_O. All tubes were incubated at 50 °C for 20 min. 2.5 ml of prepared TCA was added to all tubes. All tubes were centrifuged at 3000 rpm for 10 min. 50μl upper layer from all tubes was taken, mixed with 50 μL distilled water in wells. 10 μL ferric chloride was added to each well except the sample blank wells. All wells were left to stand for 30 min at 25 °C min (the test was carried out in triplicate in each mixture using a microplate). The absorbance of the blue ferrous complex was measured at 630 nm.

**Calculation**

The reducing capacity activity of vitamin E/oil was estimated from the following formula

**Ferric reducing capacity inhibition (%) = [(AC- Ast or AS)/AC] *100**

Where;

AC: The mean absorbance of the negative control.

Ast: The mean absorbance of vitamin E - the mean absorbance of blank control.

AS: The mean absorbance sample - the mean absorbance sample blank.

100: The percentage of inhibition.

The reducing capacity of ascorbic acid and wheat germ oil was expressed as **EC50.** Where **EC50** value (mg/mL) is the effective concentration at which 50% of the ferric ion was reduced. It was calculated by interpolation from the graph of reducing capacity percentage against sample concentration using linear regression equations (for ascorbic acid, oil)

**Y= ax + c**

From the inhibition curve, **EC 50** was calculated

Y= 50

a= slope

X= effective concentration of the sample at which 50% of the ferric ion was reduced

C= intercept

- 1. **Entrapment efficiency (EE%)**

The concentration of free unentrapped WGO in the filtrate was determined after separation of NLCs using an ultrafiltration technique. Briefly, 5 mL of the formulation was added to the upper chamber of the ultracentrifugal concentrator (SartoriusTM Vivaspin 6TM, MWCO 100,000). The Vivaspin tubes were centrifuged for 1 h at 6000 rpm at room temperature. An aliquot of filtrate was analyzed using a UV–Vis spectrophotometer at a wavelength of **212** nm to determine the concentration of the free oil. Samples were measured in triplicate and represented as mean value ± SD. The concentration of entrapped WGO was calculated as the difference between the total amount of oil added to the formulation and the amount of unentrapped free oil according to the following equation:

$$\boldsymbol{EE\%=}\frac{\mathbf{W}_{\boldsymbol{T}}\boldsymbol{-}\mathbf{W}_{\boldsymbol{F}}}{\mathbf{W}_{\boldsymbol{T}}}\boldsymbol{\times100}$$

Where WT is the total amount of oil added to the NLCs, and WF is the free unentrapped oil in the supernatant.

**Calibration curve of wheat germ oil**

| **Concentration mg/mL** | **Absorbance at 212 nm** |
| --- | --- |
| **1** | **0.065±0.012** |
| **2** | **0.098±0.017** |
| **5** | **0.248±0.013** |
| **7.5** | **0.344±0.012** |
| **10** | **0.477±0.014** |
| **12.5** | **0.559±0.019** |
| **15** | **0.654±0.011** |

The results are expressed as (Mean ± SE,n=3)

**Figure1:** Calibration curve of wheat germ oil

- 1. **Determination of superoxide dismutase (SOD) activity**

Superoxide dismutase (SOD, EC 1.15.1.1) was assayed in lung and spleen tissue extracts. This assay relies on the ability of the enzyme to inhibit the phenazine methosulphate-mediated reduction of nitroblue tetrazolium dye. Superoxide dismutase (SODs) are metalloenzymes that catalyze the dismutation of the superoxide anion to molecular oxygen and hydrogen peroxide and thus form a crucial part of the cellular antioxidant defense mechanism.

2O_2_^•^+2H+ + SOD→ H_2_O_2_ + O_2_

Three types of SODs have been characterized according to their metal content: copper zinc (Cu/Zn), manganese (Mn), and iron (Fe). SOD is widely distributed in both plants and animals. It occurs in high concentrations in the brain, liver, heart, erythrocytes, and kidneys. In humans, there are three forms of SOD: cytosolic Cu/Zn-SOD, mitochondrial Mn-SOD, and extracellular SOD. Extracellular SOD is found in the interstitial spaces of tissues and also in extracellular fluid, accounting to the majority of the SOD activity in plasma and lymph fluids. One mL of working reagent consisted of R1 + R2 + R3 in ratio of (10+1+1 mL) mixed immediately before use, R1, composed of phosphate buffer pH 8.5, 50 mmol/L, R2, nitroblue tetrazolium (NBT) 1mmol/L, and R3, NADH 1 mmol/L; mixed and added to 100μL of sample or 100μL of distilled water for control. 100μL of phenazine methosulphate (PMS) 0.1 mmol/L was added to initiate the reaction. The increase in absorbance was measured at 560 nm for 5 min for the control (ΔA control) and for the sample (ΔA sample) at 25°C. SOD activity was calculated as follows:

IU/ g tissue = % inhibition × 3.75

3.75= rate of PMS depletion per one minute

- 1. **Assay of thiobarbituric acid reactive substances (TBARS)**

One mL of homogenate was added to 2 mL of 7.5% trichloroacetic acid and mixed. The mixture was centrifuged at 1,000 xg for 10 min. 2 mL of supernatant added to 1 mL of 0.7 % 2- thiobarbituric acid. After boiling for 10 min, the reactants were cooled and TBARS were measured at 532 nm. An extinction coefficient of 156.000 mol-1cm-1 was used for calculation.

TBARS Concentration in tissue (nmol/g tissue) =

= Molar extinction coefficient 0.156 μmol-1/cm.

A = Sample absorbance

- 1. **Determination of reduced glutathione (GSH)**

The method based on the reduction of 5,5' dithiobis (2 - nitrobenzoic acid) (DTNB) with glutathione (GSH) to produce a yellow compound. The reduced chromogen directly proportional to GSH concentration and its absorbance can be measured. 500 μL of trichloroacetic acid (TCA) 500 mmol/L was added to 500 μL of tissue extract or 100 μL of blood, the mixture was mixed well, let to stand for 5 min. at room temperature and centrifuged at 10,000 xg for 15 min.; 500 μL of the supernatant was added 1000 μL of potassium phosphate buffer, 100 mmol, pH 7.0. The reaction was initiated by adding 100 μL DTNB 1.0 mmol/L the mixture was mixed well and the absorbance was measured after 5-10 min. at 405 nm against the blank; linearity is up to 120 mg/dL

A sample x 66.66

Gram tissue used

GSH concentration in tissue (mg/g tissue) =

A Sample: Absorbance measure of Sample

66.66 =Extinction value

- 1. **Determination of Glutathione-S-transferase (GST)**

Glutathione-S-transferase (GST; EC 2.5.1.18) as the follow: the interaction mixture was contained of 30 μg protein of the supernatant fraction. 0.1 mL of the enzyme source (30 μg/mL) was added to 0.2 mL of reduced glutathione (0.5 mmol/L), 2 mL of sodium phosphate buffer pH 7.4, the mixture was incubated at 37°C for 5min, then the reaction was initiated by adding 0.2mL of 1-chloro-2,4-dinitrobenzene (CDNB,1.5 mmol/L) and incubated at 37°C for another 5 min; the reaction was terminated by addition of 0.2 mL of trichloroacetic acid solution (33% W/V). After centrifugation, the CDNB conjugate was measured spectrophotometrically in the supernatant at 340 nm. Calculation was made using a molar extinction coefficient of 9.6 mmol-1cm-1. A unit of enzyme activity is defined as the amount of enzyme that catalyzes the formation of 1 μmol of CDNB conjugate/mg protein/min under the assay conditions.

GST activity (IU/g tissue) = [(A sample Δ340/min)/ 0.0096] X (V / Vs) X sample dilution

Molar extinction of CDNB is 0.0096 μmol-1/cm.

A sample = Sample absorbance

V = Total volume of reaction

Vs = volume of sample

- 1. **Determination of glutathione peroxidase (GPx)**

Glutathione Peroxidase (GPx) (GPx; EC 1.1.1.9) was determined as the follow: the assay is an indirect measure of the activity of c-GPx. Oxidized glutathione (GSSG), produced upon reduction of organic peroxide by c-GPx, and is recycled to its reduced state by the enzyme glutathione reductase

R-O-O-H + 2 GSH R-O-H +GSSG +H_2_O

GSSG + NADPH + H^+^ 2GSH + NADP^+^

The oxidation of NADPH to NADP^+^ is accompanied by a decrease in absorbance at 340 nm providing a spectrophotometry means for determining GPx activity. The molar extinction coefficient for NADPH is 6220 mol^-1^ cm^-1^ at 340 nm. To assay c-GPx, a cell of tissue homogenate is added to a solution containing glutathione, glutathione reductase, and NADPH. The enzyme reaction is initiated by adding the substrate, hydrogen peroxide, and recorded at 340 nm. The rate of decrease in the A 340 is directly proportional to the GPx activity in the sample.0.01ml of sample was added to 1mL of buffer solution consisted of, potassium phosphate Buffer, 50 μmol,/L pH 7.0 and 0.1% Triton X-100 reagent; then 0.1mL of NADPH reagent consisted of Glutathione (GSH) 24 μ mol, glutathione reductase enzyme ≥ 12 IU and Β-nicotinamide-adenine dinucleotide 4.8 μmol was added, the reaction is initiated by adding substrate solution, consisted of hydrogen peroxide 6%, (dilute 100 times before use). Decrease in absorbance was recorded against blank solution using 1 cm thick cell at wavelength 340 nm over a period of 3 min since the initiation of the reaction. The following equation was used for calculating the enzyme activity:

GPx activity (IU/ g tissue) = ×121

ΔA sample/ min

0.00622

ΔA (Sample): Change in measure absorbance of sample

0.00622 = extinction coefficient

121 = dilution factor

- 1. **Determination of glutathione reductase (GR)**

Glutathione reductase (GR) (E.C.1.6.4.2) was determined as the follow: the test principle is that GR catalyze the reduction of glutathione (GSSG) in the presence of NADPH, which is oxidized to NADPH+.

NADPH + H^+^+ GSSG NADPH^+^+ 2GSH

Fifty μL of sample was added to 1mL of buffer, composed of phosphate buffer 100 mM/L and 1 mM/L EDTA, pH 7.5; then 100 μL of GSSG, 50 mmol / L, reconstituted with 5 ml of buffer was added. Once adding NADPH reagent composed of 2mmol/L NADPH reconstituted with 5 mL of distilled water, the decrease of absorbance was measured over a period of 5 min at 340 nm. The following equation was used for calculating the enzyme activity:

GR activity (IU/ g tissue) = 4019 ×∆A

∆A (Sample): Change in measure absorbance of sample

4019 = extinction value

- 1. **Immunohistochemical studies**

EGFR proteins were carried out using immunohistochemistry protocol of the Striptoavidin Biotin Complex (SABC) (A universal kit (SABC – HRP) reagent and DAB stain) from Thermo Fisher – USA). The formalin fixed paraffin sections (FFPE) were cut 5 µm-thick, placed on coated glass slides and proceed for immunostaining. The slides were deparaffinized, rehydrated and rinsed in distilled water. Slides placed in large amount of antigen retrieval (citrate buffer pH 6) in an oven at 95 °C for 20 minutes. Then washed in phosphate buffer saline (PBS) twice 5 minutes each. Endogenous peroxides activity was quenched using 3.0% H_2_O_2_ in PBS. The slides washed in PBS twice 5 minutes each. The incubated with serum blocking reagent for 30 minutes and washed in PBS twice 5 minutes each, then incubated with primary antibodies of TNF-α and GP88 over night at 4°C in the humidified chamber. Negative control slides were incubated without adding primary antibody. The 2nd day the slides allowed to reach the room temperature and then washed in PBS twice 5 minutes each and incubated with conjugated SABC-HRP reagent for 30 minutes in humidified chamber at room temperature, then washed in PBS twice 5 minutes each. The slides stained with chromogen DAB, the chromomeric reaction turned the epitome sites brown. The slides rinsed in distilled water, stained with hematoxylin as nuclear counter stain, and then rinsed in running tap-water. Finally, Sections were dehydrated, cleared and mounted. The (EGFR) expression indicated by dark brown stain labeling the tissue and the blue color indicated to the negative reaction. The semi quantitative evaluation of the immunohistochemically results of both primary antibodies done according to the intensity of stain (+1 weak, +2 moderate, +3 strong, +4 intense).
